# Block Poly(carbonate‐ester) Ionomers as High‐Performance and Recyclable Thermoplastic Elastomers

**DOI:** 10.1002/anie.202210748

**Published:** 2022-10-21

**Authors:** Georgina L. Gregory, Gregory S. Sulley, Joost Kimpel, Matylda Łagodzińska, Lisa Häfele, Leticia Peña Carrodeguas, Charlotte K. Williams

**Affiliations:** ^1^ Chemistry Research Laboratory University of Oxford 12 Mansfield Road Oxford OX1 3TA UK

**Keywords:** Ionomers, Epoxide and Anhydride Ring-Opening Copolymerization, Sustainability, Thermoplastic Elastomer, Polyesters

## Abstract

Thermoplastic elastomers based on polyesters/carbonates have the potential to maximize recyclability, degradability and renewable resource use. However, they often underperform and suffer from the familiar trade‐off between strength and extensibility. Herein, we report well‐defined reprocessable poly(ester‐*b*‐carbonate‐*b*‐ester) elastomers with impressive tensile strengths (60 MPa), elasticity (>800 %) and recovery (95 %). Plus, the ester/carbonate linkages are fully degradable and enable chemical recycling. The superior performances are attributed to three features: (1) Highly entangled soft segments; (2) Fully reversible strain‐induced crystallization and (3) Precisely placed Zn^II^‐carboxylates dynamically crosslinking the hard domains. The one‐pot synthesis couples controlled cyclic monomer ring‐opening polymerization and alternating epoxide/anhydride ring‐opening copolymerization. Efficient convresion to ionomers is achieved by reacting vinyl‐epoxides to install Zn^II^‐carboxylates.

## Graphical abstract

Poly(carbonate‐*b‐*esters) were designed that undergo strain‐induced crystallization in the polycarbonate rubbery mid‐segment and are reinforced by reversible ionic interactions in the outer semi‐aromatic polyester blocks. The thermoplastic elastomers show impressive tensile strength, high elastic recovery and are degradable in aqueous alkali media or chemically recyclable to monomer.

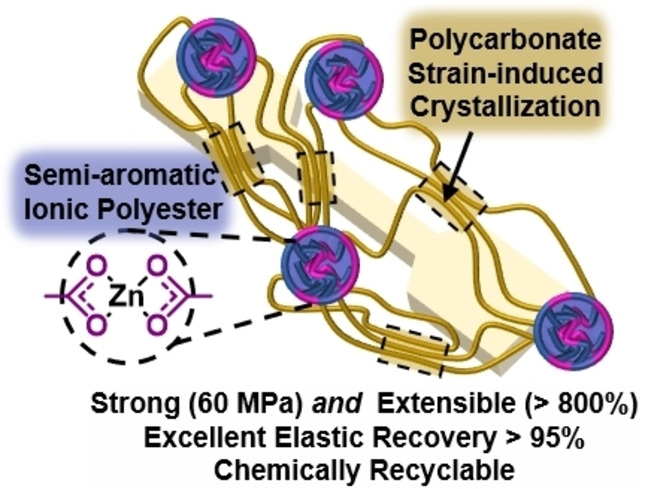

## Introduction

Thermoplastic elastomers (TPEs) are indispensable components in the automotive, healthcare and electronics industries, with a predicted worldwide production of 5.55 Mt in 2026.^[1]^ Exploiting physically crosslinked networks, they combine the elasticity of rubbers with the thermal (re)processability of plastics. These features make TPEs attractive substitutes for thermoset rubbers, where permanent chemical crosslinking hinders reprocessing and recycling.^[2]^ Despite these promising attributes, TPE performances and sustainability still need attention. One long‐standing challenge is developing elastomers with high tensile strength without compromising elasticity and elastic recovery.^[3]^ Sustainable polymer design must also prioritize use of renewable raw materials, apply scalable, efficient and reproducible polymerizations and polymer chemistry amenable to recycling and, ultimately, complete degradation.^[4]^ Polycarbonates and polyesters are attractive in this regard since many monomers are bio‐based, and the polymer‐monomer equilibria can be manipulated to recycle the products back to monomers or to ensure total chain degradation by solvolysis.^[5]^ Although prior work has demonstrated the potential for polyester‐based TPEs, often exploiting bio‐based polylactide (PLA), these materials do not yet match the high‐performance of chemically crosslinked rubbers, polyurethanes (TPUs) or copoly(ester‐ethers).^[6]^

ABA‐block polymers, where *A*=hard (high glass transition temperature, *T*_g_) and *B*=soft (low *T*_g_) blocks, deliver physically crosslinked TPE networks through microphase separation. Typically spherical/cylindrical A‐domains dispersed in a rubbery B‐matrix endow elastomer characteristics.^[7]^ Hillmyer and co‐workers have pioneered aliphatic block polyester elastomers using controlled cyclic ester ring‐opening polymerizations (ROP).[^6c^, ^8^] The best performing materials showed enhanced physical crosslinking through semi‐crystalline or stereo‐complexed PLLA/PLDA A‐domains dispersed in various bio‐derived soft polyesters.[^6f^, ^8b^] These materials achieved impressive ultimate tensile strengths (UTS) up to 30–35 MPa and elongation at breaks (ϵ_b_) from 800–1200 %.^[8b]^ Incorporating hydrogen‐bonding and ionic interactions could drive these performances up further.^[9]^ However, it is very difficult to achieve these substitutions in conventional polyester chemistry. Accordingly, ionomers are rare in polyester/carbonate TPEs.^[10]^

An alternative controlled polymerization route to polyesters is epoxide/anhydride ring‐opening copolymerization (ROCOP). This method can enchain a wide range of commercial monomers to make flexible or rigid polyesters.^[11]^ Many of these monomers are or could be bio‐sourced such as phthalic anhydride (PA) from corn stover.^[12]^ Nevertheless, polyester ROCOP is still underexplored in TPEs.^[13]^ One major advantage of ROCOP is that it functions efficiently using substituted and aromatic monomers, which can be challenging in cyclic monomer ROP.^[14]^ For example, PA/cyclohexene oxide ROCOP yields a semi‐aromatic polyester with *T*_g_>100 °C, significantly higher than that for PLA (*T*_g_=50–60 °C). In our recent work, this led to TPEs with high upper use temperatures (≈140 °C) when combined with soft poly(ϵ‐decalactone) mid‐segments.^[13a]^ The ability to use several vinyl‐substituted epoxides in ROCOP also allows functional groups to be readily introduced (e.g. using thiol‐ene reactions).^[15]^ The vinyl groups can be easily converted into carboxylic acid and metal carboxylate moieties.^[16]^ In contrast to rubbers, these groups are regularly placed along the alternating ROCOP backbone leading to more precisely controlled networks.^[17]^

Here, we combine poly(trimethylene carbonate) (PTMC) as the soft block with Zn‐ionomer polyesters from ROCOP (A‐blocks). PTMC has been scarcely explored in TPE applications, despite several appealing properties.^[18]^ It is an attractive soft segment due to its low *T*_g_ (−12 to −18 °C) and high chain entanglement (molecular weight between entanglements, *M*_e_=2.7 kg mol^−1^).^[19]^ More entangled polymer networks (low *M*_e_) typically correlate with higher TPE UTS.[^8b^, ^20^] Earlier reports also suggest that amorphous PTMC can crystallize under strain.^[19]^ Such strain‐induced crystallization (SIC) behaviour is directly associated with the superior mechanical performances of rubbers.^[21]^ This also includes greater crack growth resistance and fatigue behaviour. A challenge however, is to achieve fully reversible SIC to ensure excellent elastic recovery.^[22]^ PTMC is best synthesised by the ROP of TMC, which in turn can be bio‐sourced by coupling 1,3‐propanediol (a fermentation product of carbohydrates)^[23]^ with carbon dioxide.^[24]^ Here, in one‐pot, TMC ROP is switched to PA/vinyl‐cyclohexene oxide (vCHO) or limonene oxide (LO) ROCOP to deliver ABA‐block polymer TPEs. These all‐polyester/carbonate ionomers reach the high‐performances of TPUs and chemically crosslinked rubbers. Furthermore, the materials are re‐processable, chemically recyclable and/or fully degradable at end‐of‐life.

## Results and Discussion

Synthesis of the target poly(ester‐*b*‐carbonate‐*b*‐esters) requires controlled cyclic carbonate ROP and alternating epoxide/anhydride ROCOP. Our research group, and others, have reported upon a form of switchable catalysis whereby a single catalyst is directed between different polymerization mechanisms by the presence/absence of monomers.^[25]^ This method may improve upon sequential epoxide/heteroallene ROCOP or sequential heterocycle ROP because it obviates multiple catalyst rate balancing, intermediate purification and careful management of monomer reactivity ratios. In switchable polymerizations, epoxide/anhydride ROCOP occurs selectively over cyclic monomer ROP.^[26]^ Accordingly, first TMC ROP was investigated using an active and highly controlled ROCOP catalyst: [LZn_2_Ph_2_] (L=macrocyclic ligand, Scheme S1 for catalyst structure).^[27]^ This organometallic catalyst features phenyl ligands which react rapidly with alcohols to form the initiator in situ, ensuring high end‐group fidelity and clean formation of triblock structures. To prepare the hydroxyl‐telechelic B‐block, 1,4‐benzenedimethanol (BDM) was used as the alcohol initiator. Polymerization control was characterized by a linear increase in PTMC molar mass (*M*_n_) with monomer conversion whilst maintaining narrow molar mass distributions (dispersity, *Ð*<1.3) (Figure S1). In contrast, TMC ROP using commercial Sn(Oct)_2_ catalyst typically affords polydisperse PTMC (*Ð*≥1.7).^[19]^ PTMC with high *M*_n_ of 167 kg mol^−1^ (*Ð*=1.34) were accessed by conducting polymerizations in THF at room temperature (RT) using low catalyst loadings (0.03 mol %, TOF≈3000 h^−1^). Polymerizations were also successful in the melt (130 °C) reaching high conversions in <10 mins at 4000 equivalents of TMC to catalyst (Table S1).

**Figure 1:**
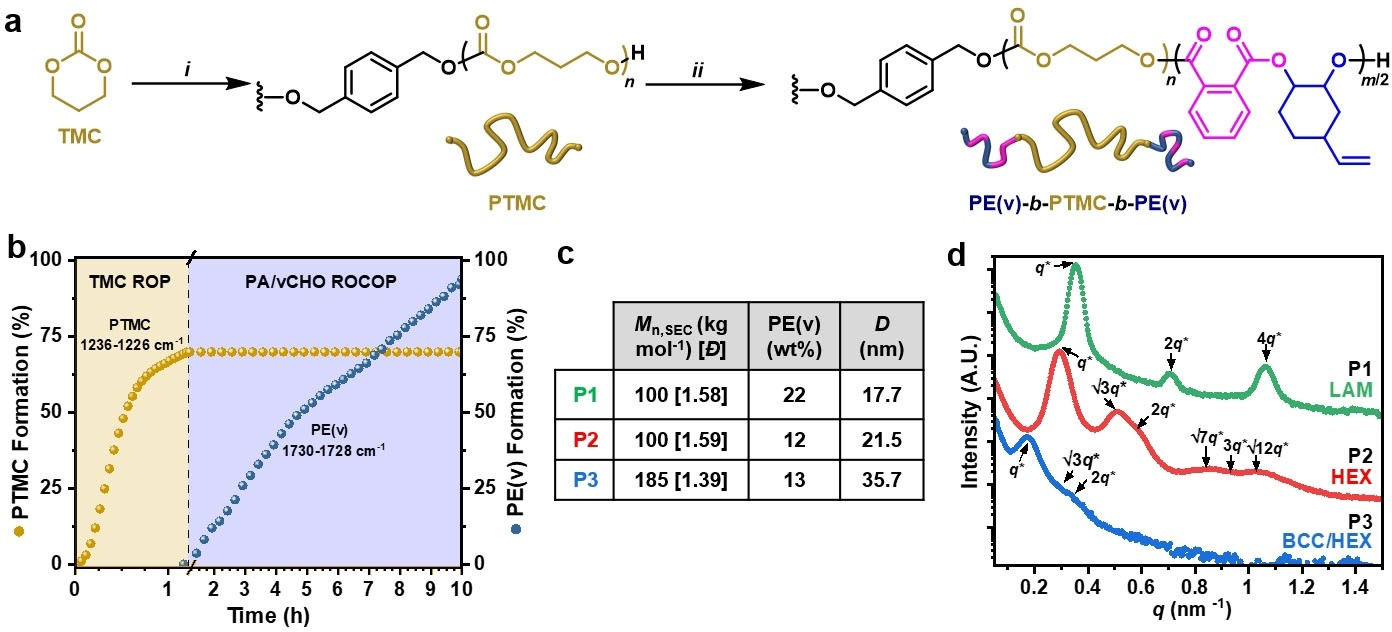
a) *i*=TMC ROP in THF (1 M) at RT, bifunctional initiator=1,4‐benzenedimethanol. *ii*=PA/vCHO ROCOP, 80 °C (Table S1–2). b) In situ React IR monitoring of PTMC formation and “switch” to polyester, PE(v) formation on adding PA. c) Synthesized ABA polymers, **P1**–**P3** with different wt % PE(v) and overall *M*_n,SEC_ (*Đ*=*M*_w_/*M*_n_ vs PS standards). Domain spacing (*D*) from SAXS (=2π/q*) where q*=principal scattering peak. d) RT SAXS profiles of **P1**–**P3**. HEX=hexagonal packed cylinders, LAM=lamellar and BCC=body‐centered cubic spherical morphology.

Triblock polymers were then formed by adding PA/vCHO to the reaction to yield alternating polyester end‐blocks (henceforth referred to as PE(v)) (Figure 1a). At this stage, the reaction temperature was increased to 80 °C to enhance rates. In situ ATR‐IR spectroscopy showed that once the anhydride was added, shown at 70 % TMC conversion, there was an immediate switch in the polymerization mechanism from TMC ROP to vCHO/PA ROCOP (Figure 1b). Monitoring the reaction by regular aliquot analysis (NMR spectroscopy) also clearly demonstrates the high monomer selectivity (Figure S2). The selective formation of PE(v)‐*b*‐PTMC‐*b*‐PE(v) was established using several other techniques (Figures S4–S7).^[28]^ Polymer end‐group analysis showed only PE(v) end‐groups without any contaminating signals for the PTMC mid‐blocks. DOSY NMR spectroscopy showed a single diffusion coefficient for all signals, whereas a mixture of PTMC and PE(v) gives multiple diffusion coefficients. Finally, aliquot analysis using SEC shows the steady increase in molar mass throughout the reaction and retention of narrow, monomodal molar mass distributions. Three polymers, **P1**–**P3** (Figure 1c, Table S2), were identified as desirable TPE compositions featuring <30 wt % PE(v) and PTMC domains approaching or exceeding chain entanglement (100–200 kg mol^−1^).^[19]^

Small‐angle X‐ray scattering (SAXS) profiles from films of **P1**–**P3** supports the formation of phase‐separated and hence physically‐crosslinked networks (Figure 1d). These films were prepared (by solvent casting from THF) to exactly represent the mechanically tested samples. **P2** with overall *M*_n_ 100 kg mol^−1^ at 12 wt % PE(v) exhibits a principal scattering peak (q*) and several higher‐order peaks at positions *q*/q* of √3, √4, √7, √9 and √12, consistent with the morphology of hexagonally packed cylinders of PE(v) in a PTMC matrix. **P1** with nearly twice the polyester content (22 wt %) at the same overall *M*_n_ exhibits *q*/q* at 2, 3, 4 and 5 (see Supporting Information for latter peaks), characteristic of a lamellar morphology.

**Figure 2:**
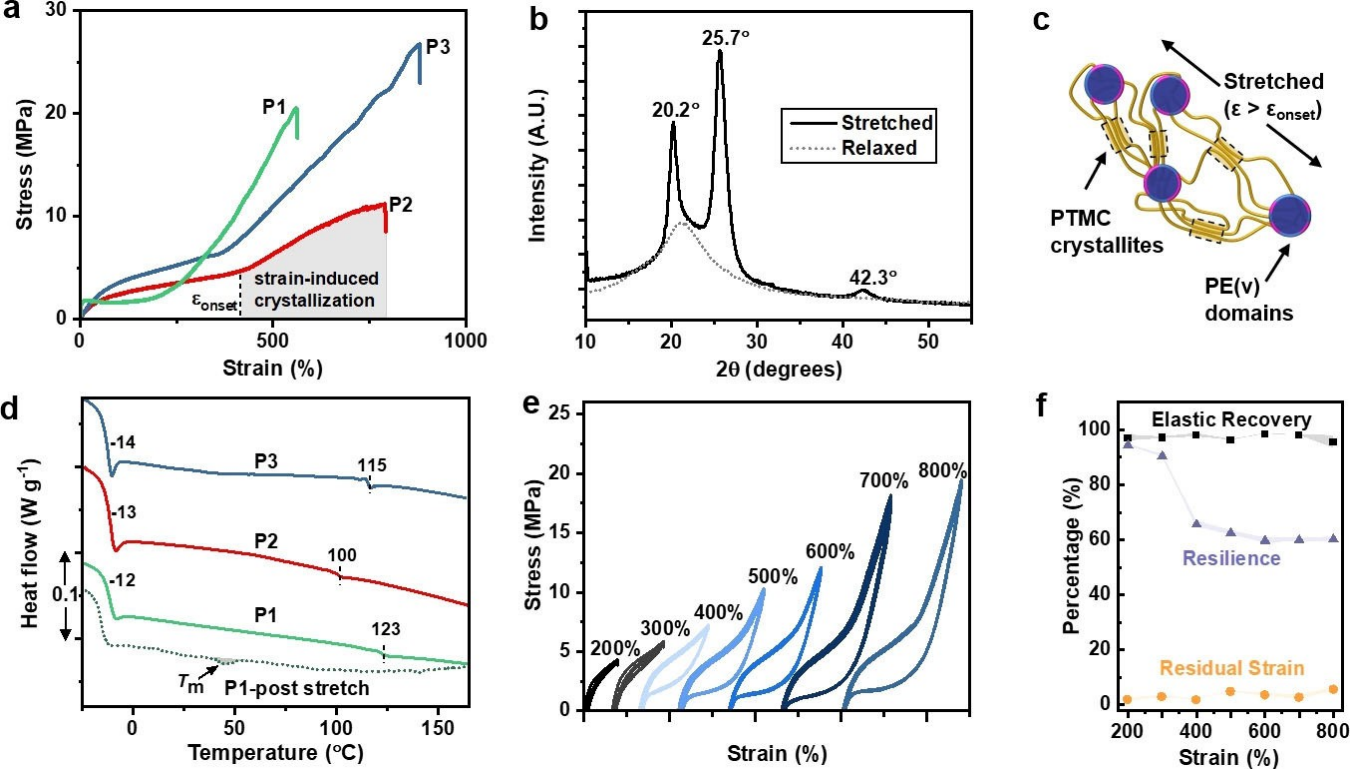
a) Tensile stress‐strain curves. b) Powder XRD pattern of **P3** in the stretched (>400 % strain) and relaxed states. c) Schematic showing strain‐induced crystallization. d) DSC curves. **P1** (dotted) was measured immediately after tensile testing (the sample was rapidly cooling to −80 °C before heating to 200 °C at 10 °C min^−1^). e) Cyclic tensile testing for **P3** to different % strains, *N*=10. f) Corresponding elasticity properties.

**P1**–**P3** were uniaxially strained at a 10 mm min^−1^ strain rate. The stress‐strain curves revealed a clear up‐turn (deviation from linearity), typically at 200 or 400 % strain (Figure 2a). This rapid increase in modulus enhances the polymers′ UTS, with values approaching 30 MPa. The behavior is attributed to strain‐induced crystallization (SIC) in the PTMC domains and requires sufficiently high molar mass for effective chain alignment (Figure S8a, Table S3).^[19]^ X‐ray diffraction (XRD) measurements performed on **P3** in the stretched state (>400 % strain) confirmed the formation of crystallites. Reflections at 20.2° and 25.7°, with a smaller peak at 42.3° is consistent with semi‐crystalline PTMC (Figure 2b).^[29]^ Based on the relative areas of the crystalline and amorphous regions, the degree of crystallinity (χ_c_) of the stretched film was estimated at ≈40 %. The sample was allowed to relax freely, after which only a broad XRD peak was observed, attributed to the sample returning to an amorphous state. Consistent with this, DSC analysis immediately after stretching showed no residual crystallinity but only the two glass transitions observed in pristine samples. These correspond to the separate amorphous PTMC (*T*_g_ −12 to −15 °C) and PE(v) phases (*T*_g_ 100–123 °C). A melting endotherm at *T*_m_ ≈40–50 °C would be expected if SIC PTMC structures were retained.^[29]^ In contrast, it was possible to ′trap′ PTMC crystallinity in **P1** after stretching it by rapid cooling (*T*_m_ 44.7 °C, Δ*H* 0.43 J g^−1^) (Figure 2d). We posit this is because **P1** features more PE(v) (22 wt %) and shows a phase‐separated lamella morphology. The SIC melting transition disappears during subsequent heating cycles.

The semi‐aromatic polyester content influenced the PTMC SIC behavior. An earlier crystallization onset strain (ϵ_onset_) was seen for **P1** over **P2**, with the former having twice the PE(v) content but the same overall *M*_n_ (100 kg mol^−1^). The modulus of the SIC segment also decreases by ≈1/3 with reduced hard block content. **P3** has the greatest overall *M*_n_ (185 kg mol^−1^) and exhibits the highest UTS, most likely as the longer PTMC chains can be aligned further in the amorphous segment. For **P3**, the crystallization onset strain was somewhat dependent on strain rate, with higher strain rates (10–500 mm min^−1^) increasing it from 370 to 460 % strain (Figure S9).

Superior elastic performance, both high strength and elastic recovery, defined as (ϵ_max_‐ϵ_*m*in_/ϵ_max_×100), is conferred when SIC structures completely melt during strain release. This crystallite loss after stretching should occur even after large deformations; the inferior elastic recovery observed in many TPEs is caused by crystallite retention.^[22a]^ To investigate these aspects, samples of **P3** were subjected to cyclic tensile testing experiments to different % strains (Figure 2e, S10). Hysteresis loops were observed when repeatedly loading/unloading to strain values sufficient to cause crystallization. The percentage of energy recovered (TPE resilience) was calculated as the ratio of the area under the unloading to loading curves. For example, when **P3** is repeatedly stretched to 200 % strain, the hysteresis loss is minimal (resilience>94 %), but when stretching to 400 % strain (ϵ>ϵ_onset_), the hysteresis was more significant (resilience ≈65 %). Critically, at high deformations (ϵ≫ϵ_onset_), the elastic recovery remained high (>95 %) with minimal residual strain (<5 %). This means that PTMC crystallite formation is fully reversible and re‐forms the random coil structure during TPE retraction (Figure 2f). Similar SIC properties are observed in stretched rubber, and the effect is of interest in advanced cooling technologies.^[30]^

**Figure 3:**
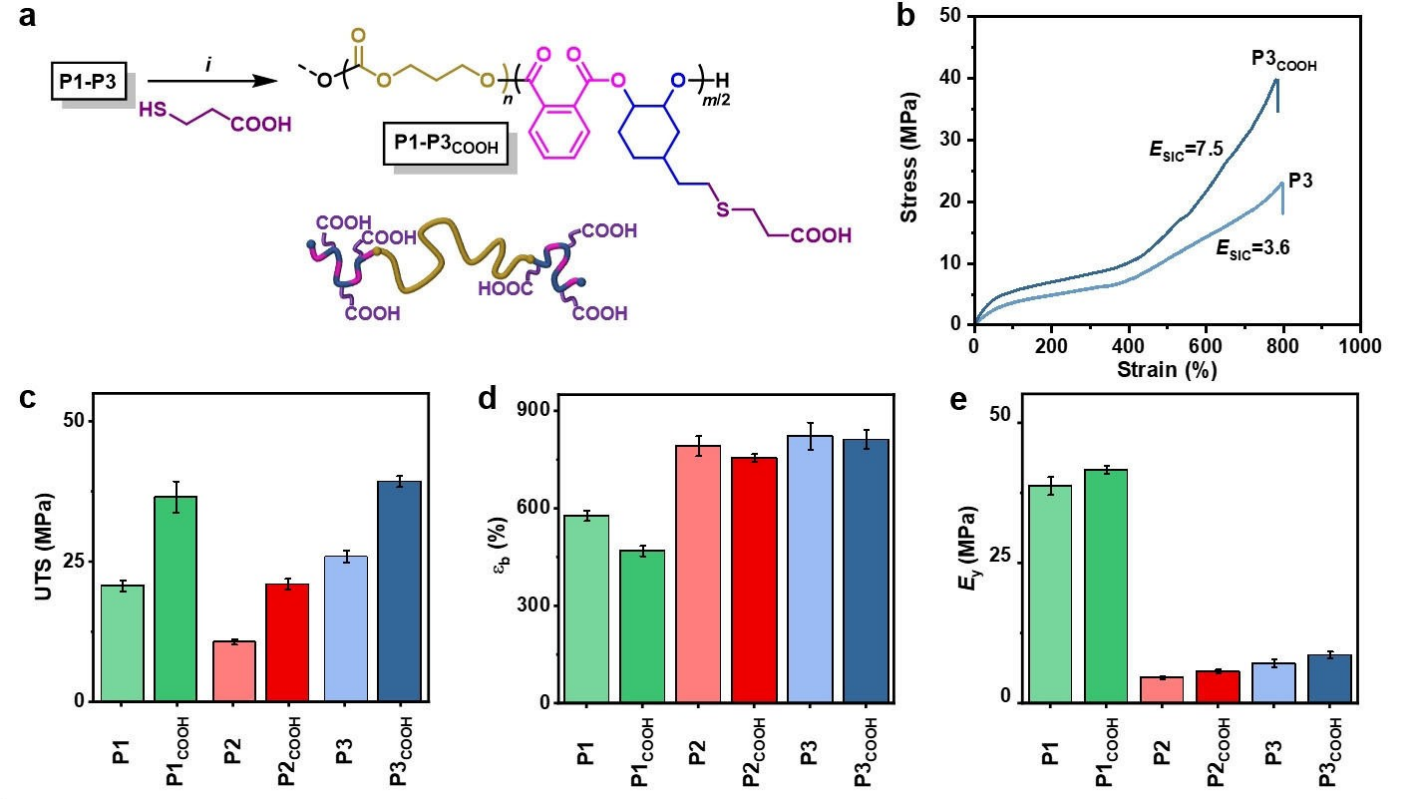
a) Reaction scheme for COOH functionalization: *i*=UV‐initiated thiol‐ene reaction with 3‐mercaptopropionic acid (see Supporting Information for details). b) Stress‐strain curves for **P3/P3_COOH_** (Figure S13 for **P1/P1_COOH_** and **P2/P2_COOH_**). c)–e) Comparison of tensile data of unfunctionalized (**P1**–**P3**) and acid functionalized (**P1**–**P3_COOH_**) TPEs (error bars=standard deviation, *N*=10).

One possible mechanical failure mechanism for TPEs is hard domain chain pull‐out.[^8b^, ^31^] Hence, we reasoned that even greater mechanical performance could be afforded by reinforcing the physical interactions in the hard segments, for example, by installing hydrogen‐bonding and/or metal‐carboxylate interactions. To investigate, regularly placed carboxylic acid functionalities were installed to the PE(v) hard domains by radical‐mediated thiol‐ene reactions (Figure 3a). The functionalized polymers (**P1**–**P3_COOH_**) were purified by precipitation from diethyl ether and characterized using ^1^H NMR spectroscopy. The spectra show the complete disappearance of vinyl proton signals at 5.8 and 4.9–5.2 ppm, and the appearance of 3‐thioether‐propionic acid substituents, at 2.5–2.8 ppm (Figure S11). SEC analyses of **P1**–**P3_COOH_** show comparable *M*_n_ and *Đ* values to the original samples, reassuring that backbone cleavage or interchain crosslinking do not occur under these reaction conditions (Figure S7). DSC analyses also confirmed that the polymer blocks remained amorphous and phase‐separated with both *T*_g_ values observed (Figure S12).

Tensile analysis of the acid‐decorated polymers showed they were distinctively stronger than the vinyl‐substituted precursors (Figures 3b–e, S13–15). In all cases, the UTS was nearly double that of the unmodified TPE, and with minimal decrease to ϵ_b_ (Table S3–4). For example, **P3_COOH_** showed average UTS of 39.3 MPa and ϵ_b_ of 812 % compared to 25.9 MPa and 822 % for **P3**. These improved properties are due to a steeper gradient in the SIC region of the stress‐strain curves (7.5 MPa for **P3_COOH_** vs 3.6 for **P3**).

**Figure 4:**
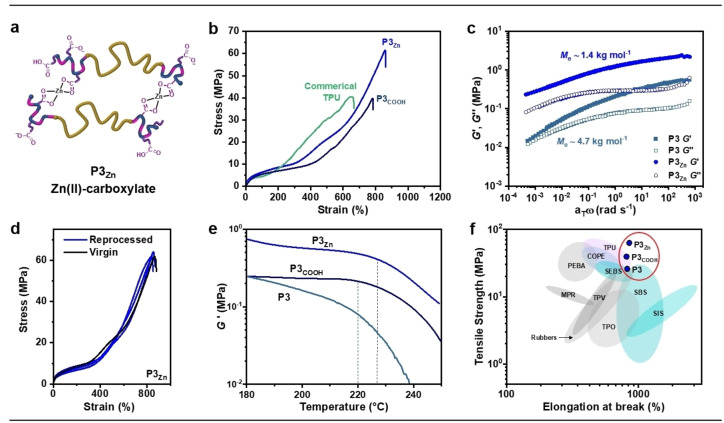
a) Schematic of **P3_Zn_**. b) Representative tensile stress‐strain curves, *N*=10. TPU=commercial thermoplastic polyurethane. c) Chain entanglement molecular weight (*M*_e_) determined from time‐temperature superposition of rheological frequency sweeps of **P3_Zn_** and **P3** at 30 °C (Figure S18 for **P3_COOH_**). d) Tensile data for 1^st^, 3^rd^ and 5^th^ thermally reprocessed films. e) Shear storage moduli (*G′*) from rheological temperature sweeps. f) Ashby plot for commercial TPEs.

Next, the carboxylic acid groups were ionized by reaction with Zn^II^ salts to form carboxylates. These ionic interactions are expected to be stronger than hydrogen bonding and thus further increase the material‘s UTS. Subsequently, the best performing sample, **P3_COOH_**, was dissolved in THF and treated with an aqueous solution of Zn^II^(acetate)_2_ (Figure 4a). The clear, colorless solution quickly became viscous, indicative of chain crosslinking, and the solution was poured into a Teflon mold. The solvent was evaporated and the film was dried in a vacuum oven (80 °C) for 72 h. ATR‐FTIR spectroscopy of the polymer film, **P3_Zn_**_,_ showed two new absorptions at ≈1597 and 1631 cm^−1^ assigned to symmetric and asymmetric zinc carboxylate stretches, respectively (Figure S16).^[32]^ Comparing the relative intensities of the Zn‐carboxylate stretches with the characteristic polycarbonate stretch (1752 cm^−1^) confirms that ≈10 mol % COOH groups (as targeted) were ionized. Despite the overall Zn content being <1 wt %, a concern was that the zinc salts might reduce TPE thermal stability. Nevertheless, thermogravimetric analysis (TGA) revealed improved high‐temperature stability for both the carboxylic acid and Zn^II^ ionomer samples compared to the original samples (Figure S17). For instance, **P3_Zn_** displayed *T*_d,5 %_≈300 °C, an increase of ≈20 °C compared to **P3_COOH_**.

Uniaxial tensile testing confirmed that **P3_Zn_** achieved a higher strength than either **P3_COOH_** or **P3** reaching a UTS of 60 MPa. Importantly, it retained its high extensibility, with ϵ_b_>800 %. The tensile strength is ≈2.5×greater than that for **P3** and 1.5×greater than **P3_COOH_** (Figure 4b). In this case, the SIC modulus remains comparable to **P3_COOH_** but provides greater elongation and, thus, higher tensile strength before break. In addition, **P3_Zn_** exhibits an increase of 4× to the Young's Modulus compared with **P3** or **P3_COOH_** (41.6 MPa for **P3_Zn_** vs 8.6 MPa for **P3_COOH_**). This increased stiffness could be consistent with greater PTMC chain entanglement or changes to phase‐separation behavior. Using rheology, the effective molecular weight between entanglements (*M*_e_) in the PTMC segment was estimated following: *M*_e_=*ρRT*/*G_N_* ^*0*^_,_ where *G_N_* ^*0*^ is the plateau in shear storage modulus (*G′*), *T* the absolute temperature (298 K), *R* the universal gas constant and *ρ* the PTMC density (1.12 g cm^−3^). Indeed, for **P3_Zn_**, *M*_e_ was estimated at ≈1.4 kg mol^−1^ compared to higher values (less entanglement) of 2.8 and 4.7 kg mol^−1^ for **P3_COOH_** and **P3**, respectively (Figure 4c, S18). These results are rationalized by Zn^II^‐carboxylate chain crosslinking being more effective at trapping entanglements in the PTMC mid‐blocks than hydrogen‐bonding interactions and ester/carbonate microphase separation.

Critically, at temperatures above 200 °C, **P3_Zn_** was successfully reprocessed multiple times (×5 tested) by compression molding without any significant impact on mechanical properties (Figure 4d). This suggests that the ionomeric interactions are reversible at higher temperatures. Rheological temperature sweep measurements conducted to 250 °C (fixed 1 Hz frequency) showed **P3_Zn_** maintained network integrity to ≈230 °C. After this, a precipitous drop in *G′* was observed, allowing for reprocessing (Figure 4e). In contrast, this drop occurred at lower temperatures for **P3** and **P3_COOH_** consistent with the presence of reinforcing Zn‐carboxylate interactions in the polyester domains in **P3_Zn_** (Figure S19). It should be noted that thermal analyses confirmed two glass transitions for **P3_Zn_** (at −16 and ≈140 °C) and a comparable rubbery plateau modulus of the pristine and reprocessed samples (Figure S20–21).

The elastomeric properties for **P3_Zn_** (and **P3_COOH_**) retained high strain recovery (>95 %) (Figure S22). Consistent with the earlier findings for **P3**, powder XRD measurements for **P3_Zn_** in the stretched (ϵ>ϵ_onset_) and relaxed states indicated that strain‐induced PTMC crystallites completely melted on retraction. Thus, the SIC properties are not affected by functionalizing the polyester end‐blocks (Figure S23). There was a slight decrease in TPE resilience (200 % strain) for the functionalized polymers: **P3_Zn_** (83 %)<**P3_COOH_** (87 %)<**P3** (94 %), which was attributed to less well‐defined phase separation (Figure S24). SAXS patterns of **P3_Zn_** and **P3_COOH_** films showed fewer higher order reflections than **P3** (Figure S25).

In this work, the key to the high TPE performances is the combination of SIC behaviour, Zn^II^‐carboxylate interactions and high chain entanglement. The latter is supported by the significantly lower UTS values reported for comparable ABA‐block polymers, employing less entangled poly(ϵ‐decalactone) (*M*_e_=6–9 kg mol^−1^)^[13a]^ or poly(γ‐methyl‐ϵ‐caprolactone) (*M*_e_≈3 kg mol^−1^)^[8b]^ mid‐segments (Figure S26). Until the introduction of polyesters made by epoxide/anhydride ROCOP, ionomeric crosslinking was not investigated for such block polyester/carbonates, perhaps due to synthetic challenges.^[33]^ Rather, stereocomplexation of opposite PLA enantiomers was explored to toughen polyester TPEs.^[34]^ In comparison, the Zn^II^‐carboxylate interactions in **P3_Zn_** result in 3 x greater UTS than similar PTMC‐based elastomers featuring stereocomplexed PLLA/PDLA hard‐domains.^[34]^ Within a commercial context, these functionalized poly(ester‐*b*‐carbonate‐*b*‐esters) lie at the high end of TPE performances (Figure 4f). They offer comparable thermo‐mechanical properties to TPUs, vulcanized rubbers and copoly(ester‐ethers) (COPEs) but differ in their chemical stability (hence the recyclability and degradability). For example, TPUs show some of the highest performances in the market but the use of toxic di‐isocyanates in their synthesis can be a drawback as well as complicating their recycling protocols.^[35]^ Encouragingly then, the stress‐strain profiles for **P3_COOH_** and **P3_Zn_** are comparable or better to a commercial TPU sample (Figure 4b).

**Figure 5:**
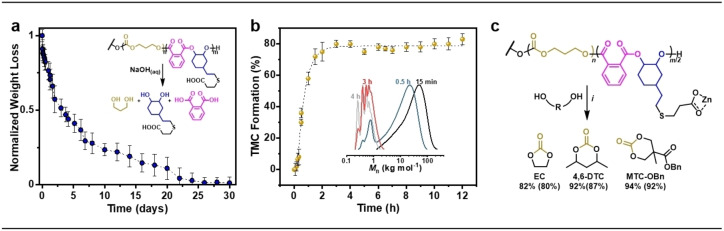
a) Hydrolytic degradation of **P3_Zn_** in NaOH_(aq_) at RT, *N*=3. b) Chemical recycling of **P1_Zn_** to TMC (NMR spectroscopy) at 80 °C in MeCN (0.1 M) using TBD catalyst (0.15 equiv) (Figure S29), *N*=3. Inset. Corresponding SEC traces of reaction aliquots confirming loss of polymer. c) Chemical upcycling to other cyclic carbonates when diol is added (3 equiv). Conversions (after 3 h) are relative to an internal standard. Isolated product yields are in brackets (Figure S30–31).

Ester and carbonate moieties also provide regular break‐points along the polymer backbone to impart end‐of‐life degradability.^[36]^ The unique degradation characteristics of PTMC have received considerable attention.^[37]^ In this study, we wanted to explore how the TPE's functionalized polyester end‐blocks might influence PTMC hydrolytic degradation. To investigate this, an accelerated degradation study was conducted by immersing discs of **P3**, **P3_COOH_** and **P3_Zn_** in 1 M NaOH_aq_. Both ionic and carboxylic acid functionalities were observed to enhance degradation (10–25 % mass loss after 24 h) compared to unmodified **P3** (<1–2 %) (Figure S27). These differences are rationalized by the hydrophilic carboxylic acid and Zn^II^ ionomers increasing water absorption (10–20 %) into the films (Figure S27). After 30 days, complete mass loss of **P3_Zn_** occurred (Figure 5a). Analysis of the degradation products by ^1^H NMR spectroscopy and LC‐MS showed clean formation of 1,3‐propanediol, 1,2‐dibenzoic acid and the diol from the functionalized epoxide (Figure S28, Table S5). These small molecules are expected to show low toxicity and be further metabolized.^[38]^

Chemical recycling of the block polymer TPEs was also investigated as transcarbonation of the PTMC could re‐form TMC monomer (Scheme S2). The polymer‐to‐monomer equilibrium should be favored under conditions of high‐dilution and temperature. 1,5,7‐Triazabicyclo[4.4.0]dec‐5‐ene (TBD) is a well‐known trans‐esterification/carbonation catalyst, and recent work has demonstrated its utility in depolymerizing commodity polyester/polycarbonates.^[39]^ Here, depolymerization was conducted in dry acetonitrile (0.1 M) with TBD catalyst (0.15 equiv) at 80 °C. After 2 min, **P1_Zn_** fully dissolved, and reaction aliquots were quenched (acetic acid) and analyzed by ^1^H NMR spectroscopy (Figure S29). After 3 h, conversion to TMC reached equilibrium (≈80 % conversion) and the monomer could be isolated from the reaction mixture in 75 % yield (Figure 5b). Alternatively, adding a diol (3 equiv), such as ethylene glycol to the depolymerization resulted in upcycling to the corresponding cyclic carbonate (Figure 5c). Ethylene carbonate (EC) isolated here in 80 % yield is a widely employed liquid electrolyte and the TMC derivatives are useful ROP monomers.^[37b]^ The concomitantly released 1,3‐propanediol is widely used in the cosmetics, pharmaceutical and polymer industries and is the precursor to reforming TMC monomer with CO_2_.^[23]^ Under these depolymerization conditions, ^1^H NMR spectroscopy of the crude reaction mixture suggests polyester conversion to the ring‐opened epoxide and phthalate ester of the diol (Figure S30–31).

To demonstrate the scope of the block TPE reported here, high‐performance, fully bio‐renewable TPEs based on PTMC and limonene oxide (LO)/PA ROCOP were also investigated. Commercial LO is currently bio‐derived from terpenes and like vCHO has an alkene functionality that can readily undergo thiol‐ene reactions. Following TMC ROP, LO/PA ROCOP was successful at 130 °C yielding the polyester hard segment, PL(v). The desired triblock polymer PL(v)‐PTMC‐PL(v) (**P4**) was isolated with *M*_n_ of 208 kg mol^−1^ (*Đ* 1.41) and 12 wt % PL(v) (Figure S32). **P4** was functionalized to install the carboxylic acid (**P4_COOH_**) and the corresponding Zn^II^ ionomer (**P4_Zn_**) using the conditions established above. The polymers showed phase‐separated structures with *T*_g_ values by DSC at −15 and 110–130 °C. The TPEs showed a high UTS of 63 MPa and ϵ_b_ of 1280 % (Table 1, Figure S33–34).

**Table 1:** Tensile data for Zn‐carboxylate modified poly(ester‐carbonate) TPEs.

	UTS [MPa]^[a]^	ϵ_b_ [%]^[a]^	*E*_y_ [MPa]^[b]^	*U*_T_ [MJ m^−3^]^[c]^
**P3_Zn_**	62.3±1.2	851±23	41.6±2.5	183±8
**P4_Zn_**	63.4±2.9	1280±50	18.3±0.5	236±15

[a] Determined by tensile testing until break (*N*≥3). [b] Young's Modulus in linear regime. [c] Tensile toughness from area under stress‐strain curve.

## Conclusion

All‐polyester/carbonate thermoplastic elastomers (TPEs) typically fall short of the high‐performances of thermoplastic polyurethanes or vulcanized rubbers. Nevertheless, they are attractive for their degradable linkages, bio‐based monomer pool and chemical recyclability. Here, block poly(ester‐carbonate) TPEs that combine high tensile strengths (60 MPa) and extensibilities (>800 %) with excellent elastic recovery (>95 %) and recyclability were reported. A single, fast di‐zinc catalyst gave the well‐defined ABA‐block polymers in one‐pot by switching from trimethylene carbonate ring‐opening polymerization to alternating vinyl‐cyclohexene oxide/phthalic anhydride ring‐opening copolymerization. When the vinyl substituents in the polyester blocks were functionalized to install Zn^II^‐carboxylate groups, the polyesters were reinforced and more chain entanglements were trapped in the poly(trimethylene carbonate) (PTMC) mid‐segments. Such functionalizations are under‐explored in block polyester/carbonate TPEs, but present a straightforward means to access superior properties. These ionomers (<1 wt % Zn) also benefit from PTMC strain‐induced crystallization that is fully recoverable after stretching. Importantly, the elastomers were also reprocessable without compromising the thermal or mechanical properties and at end‐of‐use fully degraded, under aqueous alkaline conditions to small molecules. Alternatively, efficient chemical recycling to reform TMC monomer or other useful cyclic carbonate monomers were feasible. These results motivate the continued investigation of polyester/carbonate blocks reinforced with reversible, non‐covalent interactions.

## Conflict of interest

The authors declare no conflict of interest.

1

## Supporting information

As a service to our authors and readers, this journal provides supporting information supplied by the authors. Such materials are peer reviewed and may be re‐organized for online delivery, but are not copy‐edited or typeset. Technical support issues arising from supporting information (other than missing files) should be addressed to the authors.

Supporting Information

## Data Availability

The data that support the findings of this study are available from the corresponding author upon reasonable request.
