# Supplementary material for: Block Poly(carbonate‐ester) Ionomers as High‐Performance and Recyclable Thermoplastic Elastomers
Source: Angew Chem Int Ed Engl. 2022 Oct 21;61(47):e202210748. doi: 10.1002/anie.202210748 (PMC9828403; doi:10.1002/anie.202210748)
Supplement: Supplementary file 1 — Supporting Information [file ANIE-61-0-s001.pdf]

Angewandte Chemie
Eine Zeitschrift der Gesellschaft Deutscher Chemiker

Supporting Information

Block Poly(carbonate-ester) Ionomers as High-Performance and Recyclable Thermoplastic Elastomers

G. L. Gregory, G. S. Sulley, J. Kimpel, M. Łagodzińska, L. Häfele, L. P. Carrodeguas, C. K. Williams**

Table of Contents

Experimental Details.......... 1
Methods and Reagents.......... 2
Scheme S1. ROP and ROCOP polymerization cycles.......... 3
Table S1. TMC ROP conditions.......... 3
Figure S1. TMC ROP analysis.......... 4
Figure S2. 'Switching' between TMC ROP and PA/vCHO ROCOP.......... 5
Figure S3. TMC/PA/vCHO one-pot 'switch'.......... 6
Figure S4. NMR characterization ($CDCl_3$) of PE(v)-b-PTMC-b-PE(v).......... 7
Figure S5. Polymer chain end-group analysis... 8
Figure S6. DOSY NMR spectra.......... 9
Figure S7. Size Exclusion Chromatography (SEC) of **P1-P3.**.......... 10
Table S2. Polymerization details for ABA Triblock Synthesis.......... 10
Figure S8. Additional stress-strain curves for PE(v)-PTMC-PE(v) triblock polymers.......... 11
Figure S9. Strain rate dependence of stress-strain behaviour for **P3**.......... 11
Figure S10. Cyclic tensile testing of ABA polymers to 200% strain...... 12
Figure S11. NMR characterization ($CDCl_3$) of COOH modified ABA polymer.......... 13
Figure S12. DSC traces of ABA_{COOH}.......... 14
Figure S13. Additional stress-strain curves for **P1-P2** and **P1-P2**$_{\mathbf{COOH}}$**.**.......... 14
Figure S14. Uniaxial tensile testing data for **P1-P3**$_{\mathbf{COOH}}$**.**.......... 15
Figure S15. Development of strain-induced crystallization behaviour on functionalization... 16
Table S3. Additional mechanical data for ABA and ABA_{COOH}.......... 16
Figure S16. FTIR spectroscopic analysis of triblock polymer films.......... 17
Figure S17. TGA thermograms.......... 17
Figure S18. Dynamic mechanically thermal analysis (DMTA).......... 19
Figure S19. Storage (*E*') Moduli as a function of temperature for reprocessed **P3**$_{\mathbf{Zn}}$**.**.......... 19
Figure S20. Time-temperature superposition (TTS) curves.......... 18
Figure S21. Additional Rheology Data.......... 18
Figure S22. Cyclic Tensile Testing for **P3**$_{\mathbf{Zn}}$, **P3**$_{\mathbf{COOH}}$ and **P3**... 20
Figure S23. Powder X-ray Diffraction Pattern of **P3**$_{\mathbf{Zn}}$.......... 20
Figure S24. Cyclic tensile tests at 200% strain.......... 20
Figure S25. SAXS Profiles (RT) for **P1-3**$_{\mathbf{COOH}}$ and **P1-3**$_{\mathbf{Zn}}$.......... 21
Table S4. Summary of TPE properties.......... 21
Figure S26. Ashby Plot for literature TPEs.......... 22
Figure S27. Hydrolytic degradation in aqueous alkaline media.......... 22
Figure S28. Degradation products of **P3**$_{\mathbf{Zn}}$.......... 23
Table S5. Proposed Assignment of **P3**$_{\mathbf{Zn}}$ Degradation Products in alkaline media.......... 23
Scheme S2. Proposed reaction scheme for chemical recycling of $P1_{Zn}$... 24
Figure S29. Chemical recycling of **P1**$_{\mathbf{Zn}}$**.**.......... 25
Figure S30. Chemical upcycling of **P1**$_{\mathbf{Zn}}$**.**.......... 27
Figure S31. ^{1}H NMR ($CDCl_3$) of isolated cyclic carbonates from **P1**$_{\mathbf{Zn}}$ chemical recycling... 28
Figure S32. PL(v)-PTMC-PL(v) synthesis and characterization.......... 29
Figure S33. ^{1}H NMR ($CDCl_3$) of **P4**$_{\mathbf{COOH}}$ after thiol-ene reaction.......... 30
Figure S34. Representative tensile data for **P**$_{\mathbf{4}}$, **P4**$_{\mathbf{COOH}}$ and **P4**$_{\mathbf{Zn}}$.......... 30
References.......... 30

Experimental Details

NMR Spectroscopy. ^{1}H, and $^{13}C\{^{1}H\}$ NMR were recorded on a Bruker Avance III HD 400 MHz spectrometer. DOSY spectra were recorded on Bruker Avance III HD 500 MHz spectrometer.

Size Exclusion Chromatography (SEC). Polymer (2-10 mg) dissolved in HPLC grade $CHCl_3$ (1 mL) was syringe filtered through 2 μm filters before injection into an Agilent PL GPC-50 instrument, with two PSS SDV 5 μm linear M columns heated to 30 °C. HPLC grade $CHCl_3$ was used as the eluent, at a flow rate of 1.0 mL min^{-1}, with RI detection calibrated using a series of narrow molecular weight polystyrene standards. Agilent SEC post-run program was used to analyze the data.

Differential Scanning Calorimetry (DSC). Recorded for purified polymer samples on a Mettler Toledo DSC3 Star calorimeter under a nitrogen flow (80 mL min^{-1}). Samples were cooled to -80 °C at 20 °C min^{-1}, held at this temperature for 2 minutes before being heated at the same rate to 200 °C and held for 5 minutes. Subsquent heating and cooling cycles where conducted from -80 to 200 °C at a rate of 10 °C min^{-1}. Glass transition temperatures (T_g) were determined from the midpoint of the transition in the second heating curve. The DSC was calibrated using indium and zinc standards.

Thermogravimetric Analysis (TGA). Measured on Mettler-Toledo Ltd TGA/DSC 1 system. Powder polymer samples were heated from 30 to 500 °C at a rate of 5 °C min^{-1}, under N_2 flow (100 mL min^{-1}).

Polymer films Preparation. For mechanical testing these were prepared by solvent casting from THF. The polymer was dissolved in THF before being poured into a Teflon mold and the solvent allowed to dry at room temperature for being dried in a vacuum oven at 80 °C for at least 72 hours or until no solvent was observed by NMR or TGA analysis.

Tensile Testing. Dumbbell specimens were cut according to ISO 527-2, specimen type 5B with Zwick ZCP020 cutting press (length= 35 mm, gauge length = 10 mm, width = 2 mm). Monotonic uniaxial extension experiments were carried out on a Shimadzu EZ-LZ Universal testing instrument at an extension rate of 10 mm min^{-1} (unless otherwise noted). An external camera was used to calculate the Young's Modulus, *E* within the 0.025-0.25% strain region. 10 Specimens were tested for each material. Cyclic tensile tests were conducted to a fixed % strain at a rate of 10 mm min^{-1}. 10 Cycles were measured for each specimen, 3 specimens for each sample. No rest period was allowed between cycles.

Phosphorus end-group tests. Following a literature procedure,[1] to polymer (40 mg) dissolved in $CDCl_3$ (0.4 mL) was added 40 μL of solution containing $Cr(acac)_3$ (5.5 mg) and internal standard, bisphenol A (400 mg) in pyridine (10 mL) followed by 40 μL of 2-chloro-4,4,5,5-tetramethyl dioxaphospholane.

Compression Molding. Polymer materials were compression molded into films using a Carver mini CH CE Press (5420CE.4010C00) with heated plates and a hydraulic compression press. The triblock polymers were sandwiched between two sheets of Teflon and placed into a metal frame rectangular-shaped area. Compression was conducted at 200 °C, for 15 min, under 20 MPa.

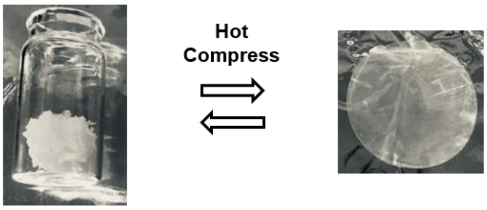

Dynamic Mechanical Thermal Analysis (DMTA). DMTA was carried out using a TA Instruments Q850 RSA-G2 Solids Analyser, using a Q18 DMA ACSIII cooling system. Highly transparent films of the triblock copolymers were prepared by solvent casting from THF. The solvent was allowed to evaporate overnight and the films were dried in a vacuum oven at 80 °C. Specimens of uniform width were cut from a solvent cast film using a Zwick ZCP020 cutting press. Samples were heated from -30 °C to 180 °C, at a rate of 3 °C min^{-1}, with a frequency of 1 Hz, 0.1 N pre-load force, and 0.1% strain amplitude. Glass transition temperatures (T_g) are reported as the peak maxima in tan(δ).

Rheology. Shear storage (*G*') and loss (*G*'') moduli were measured on a TA ARES-G2 instrument using polymer samples between 25 mm stainless steel plates. Measurements were conducted in the linear viscoelastic region as determined by amplitude sweeps. Temperature sweep experiments were conducted at a heating rate of 2 °C min^{-1}, 1 Hz frequency.

Fourier-transform infrared spectroscopy (FTIR). IR spectra were recorded on a Perkin Elmer Spectrum 100 (FT-IR) with an AT-IR crystal or a Varian FT-IR 3100 spectrophotometer (Golden Gate). All the IR measurements were performed in the reflection mode at a resolution of 4 cm^{-1}.

Attenuated Total Reflection – Infrared Spectroscopy (ATR-IR). A Mettler-Toledo ReactIR 4000 spectrometer with an MCT detector and a silver halide DiComp probe were used for in situ monitoring by ATR-IR spectroscopy. The reported plots were obtained after one or two-point baseline corrections, and the monitored bands were selected from the overlap of individual IR spectra of monomers and polymers.

Small-angle X-ray Scattering (SAXS). Polymer films (prepared as for mechanically tested films described above) were submitted to Harwell Diamond Light Source in a solid sample grid for SAXS analysis (DL-SAXS, P38 instrument). Scans (3 × 5 min) were conducted at camera lengths of 4.5 and 1 m, beam energy = 9.2 keV (using the Ga MetalJet). SAXS curves reported are an average of the 3 scans measured from data collected at 1 m. Samples were not annealed prior to testing to reflect experimental conditions of tensile testing. 2D scattering patterns were reduced to 1D using Dawn software developed at the Diamond Light Source.[2]

Powder X-ray Diffraction (XRD): Experiments were performed on polymer films in the stretched or relaxed state using a PANalytical Xpert Pro diffractometer with a Cu Kα radiation source (λ = 1.54 Å) at 40 mA and 40 kV with a step size of 0.033° 2θ, scan step time of 70 s and a scan range of 5–90° 2θ.

Methods and Reagents

Following literature procedures, trimethylene carbonate (TMC) was prepared from CO_2 and 1,3-propanediol.[3] It can also be purchased from TCI. It was purified by recrystallization from dry diethyl ether (× 3) and stored in a glovebox before use. vCHO was purchased from Sigma Aldrich, dried over CaH_2 and isolated by fractional distillation before being stored in a glovebox at -30 °C. Phthalic anhydride (99% Sigma Aldrich) was purified by extracting into anhydrous benzene (30 g in 500 mL), recrystallization from dry chloroform and sublimation (80 °C, vacuum). Anhydrous THF obtained by distillation from sodium benzophenone stills was degassed (×3 freeze-pump thaw) and stored under nitrogen over 3 Å molecular sieves. LZn_2Ph_2 (Scheme S1) was synthesized according to previous literature procedures.[4]

General Polymerization Procedure. In a glovebox filled with nitrogen, TMC (4.9 g, 48 mmol, 4000 equiv.) was weighed out and transferred to a Schlenk flask. Anhydrous THF (48 mL) was then added to dissolve the TMC ($[TMC]_0$ = 1 M) followed by BDM (7 mg, 0.05 mmol, 4 equiv.) and finally the catalyst (10 mg, 0.012 mmol, 1 equiv.). The reaction was stirred at room temperature for 1 h, whereby a noticeable increase in viscosity was observed. An aliquot was taken for ^{1}H NMR spectroscopy and SEC analysis. PA/vCHO were then added to the solution in the glovebox, and the reaction mixture was placed in an oil bath, preheated to 80 °C. PA conversion was monitored by ^{1}H NMR spectroscopy of aliquots taken at various time points. After around 48 h, the polymerization was quenched by adding benzoic acid (15 mg, 0.12 mmol, 10 equiv.) dissolved in DCM (minimum to dissolve). The polymer was isolated from the crude reaction mixture by precipitation into diethyl ether and washing with toluene as a white solid (~ 5 g).

General Procedure for Post-polymerization Functionalization. ABA triblock polymer (1 g, 0.8 mmol C=C, 1 equiv. C=C) was dissolved in degassed THF (10 mL) before adding 3-mercaptopropionic acid (140 µL, 1.6 mmol, 2.0 equiv.) and DMPA (41 mg, 0.16 mmol, 0.2 equiv.). The solution was stirred for 1 h whilst exposed to UV light. An increase in the viscosity of the solution was noted. The crude reaction mixture was then poured into a large excess of rapidly stirring diethyl ether (~ 500 mL) to precipitate the functionalized polymer as a white solid (~ 1 g). The ether was decanted off, and the polymer was washed with fresh diethyl ether (×2) before being dried under a vacuum.

General Procedure for Zn(II)-Ionomer Formation. To a rapidly stirring solution of polymer (0.2 g, 0.12 mmol COOH, 1 equiv. COOH) dissolved in THF (2 mL), was added $Zn(II)OAc_2.H_2O$ in an aqueous solution (0.1 M, 207 µL, 0.1 equiv. relative to COOH). An increase in the viscosity of the solution was noted. After 15 minutes of stirring at room temperature, the clear, colourless solution was poured into a Teflon mould and allowed to dry under ambient conditions, for 48 hours, before being dried in a vacuum oven at 60 °C.

Degradation Experiments. Polymer films (300 µm thick) were cut into discs (16 mm diameter) and submerged in 1 M NaOH solution (pH = 14). Samples were recovered at various time intervals and vacuum dried to constant mass. Mass loss was determined by gravimetric mass as: $((m_{t=0} - m_{t=x}) / m_{t=0}) \times 100$). After complete weight loss (no discernable insoluble mass remaining), the aqueous solution was analyzed by ^{1}H NMR spectroscopy (D_2O) and liquid-chromatography mass spectrometry (LC-MS) in D_2O/H_2O with formic acid to aid ionization. Experiments were conducted in triplicate. Water uptake

refers to water absorbed in the sample during experiments conducted of discs submerged in distilled water: water uptake (%) = (($w_{t=x}$ - $w_{t=0}$) / $w_{t=0}$).

Depolymerization Experiments. Polymer (100 mg, 1 equiv.) was added to anhydrous acetonitrile (0.1 M) under nitrogen, and TBD (16 mg, 0.15 equiv) added before transferring the reaction to an oil bath preheated to 80 °C. The reaction was quenched by adding acetic acid (10 equiv. relative to TBD).

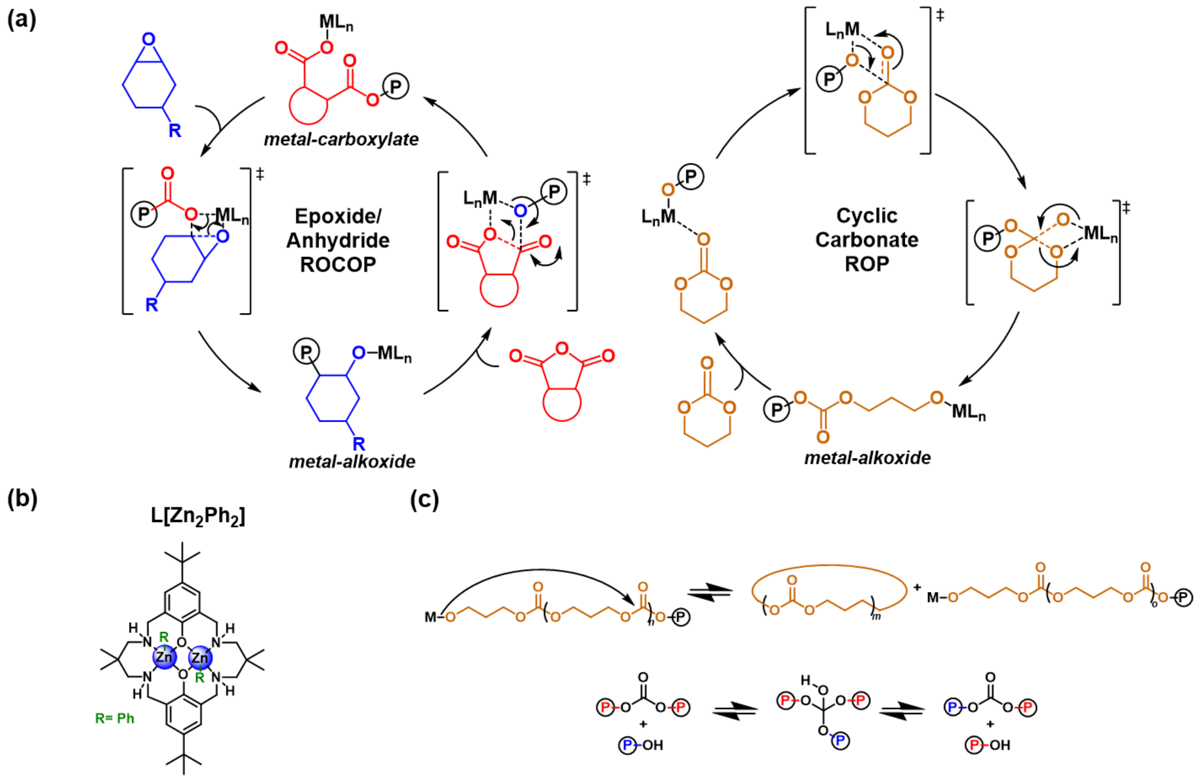

Scheme S1. ROP and ROCOP polymerization cycles. (a) Anhydride/epoxide ROCOP and TMC ROP. (b) Structure of catalyst used in this work. This catalyst has previously been shown to be highly active for ROCOP with high selectivity for perfectly alternating sequences.[3] (c) Possible side reactions (shown for polycarbonate synthesis) include backbiting and transcarbonation exchange reactions (not observed).

Table S1. TMC ROP Conditions.[a]

Solvent	T (°C)	t (h)	Conv. (%)[b]	[Cat] : [I] : [TMC]	$M_{n,calc}$ (kg mol^{-1}) [c]	$M_{n,SEC}$ (kg mol^{-1})[d]	Đ[d]
vCHO	22	1.5	96	1:4:500	12.4	20.1	1.37
vCHO*	22	1.0	n.d*	1:4:4000	100	51.0*	1.68
THF	22	1.0	78	1:4:4000	79.6	88.9	1.41
THF	22	1.0	72	1:2:4000	147	165	1.38
THF	22	1.0	76	1:2:4000	155	167	1.34
melt	130	0.2	70	1:4:4000	76.2	80.3	1.42

[a] $[TMC]_0$ = 1.0 M. Catalyst = LZn_2Ph_2. * = Higher molar mass PTMC is insoluble in vCHO and toluene solvents and precipitated from solution. [b]Conversion determined by ^{1}H NMR ($CDCl_3$) integrals of TMC (δ 4.45 ppm) and PTMC (δ 4.23 ppm). [c] Theoretical M_n based on starting monomer to initiator ratio, TMC conversion and M_{TMC} = 102 g mol^{-1}. [d]$M_{n,SEC}$ and $Đ$ determined by SEC (RI detector vs PS standards). $Đ = M_w/M_n$.

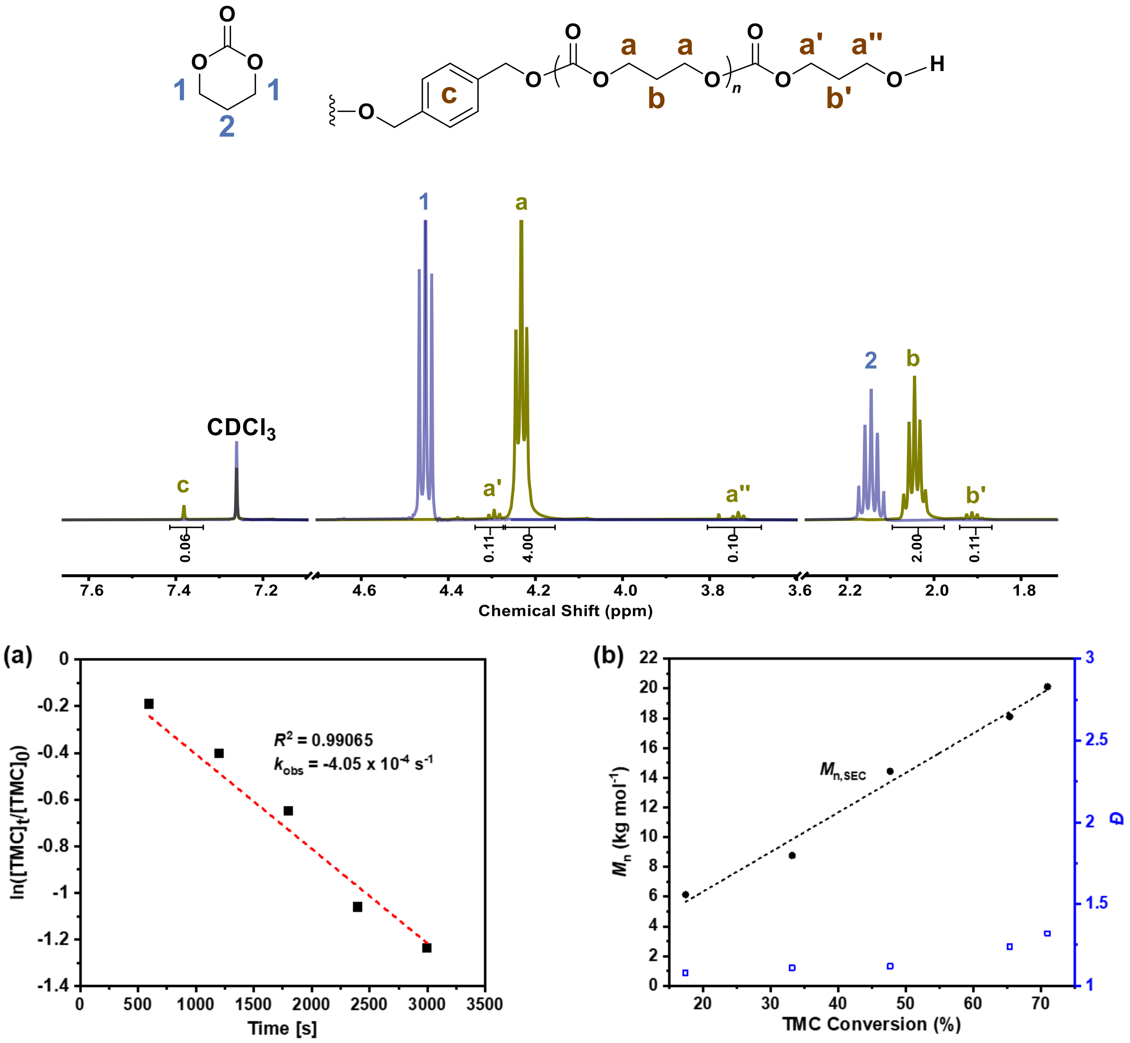

Figure S1. TMC ROP analysis. Top: Overlaid ^{1}H NMR spectra ($CDCl_3$) of TMC (blue) and purified PTMC (gold, $M_{n,SEC}$ = 7000 kg mol^{-1}). Below: (a) Kinetic plot: ln([TMC]$_t$/[TMC]$_0$) vs time using [LZn_2Ph_2] in vCHO at RT, [TMC]$_0$ = 1.0 M, [LZn_2Ph_2] : [BDM] : [TMC] = 1:4:500. There is a first-order dependence on rate with [TMC]. (b) M_n and $Đ$ vs TMC conversion with [LZn_2Ph_2] in vCHO at RT. A linear relationship between TMC conversion to PTMC with M_n whilst mainting monomodal distributions is consistent with controlled 'living' polymerization.

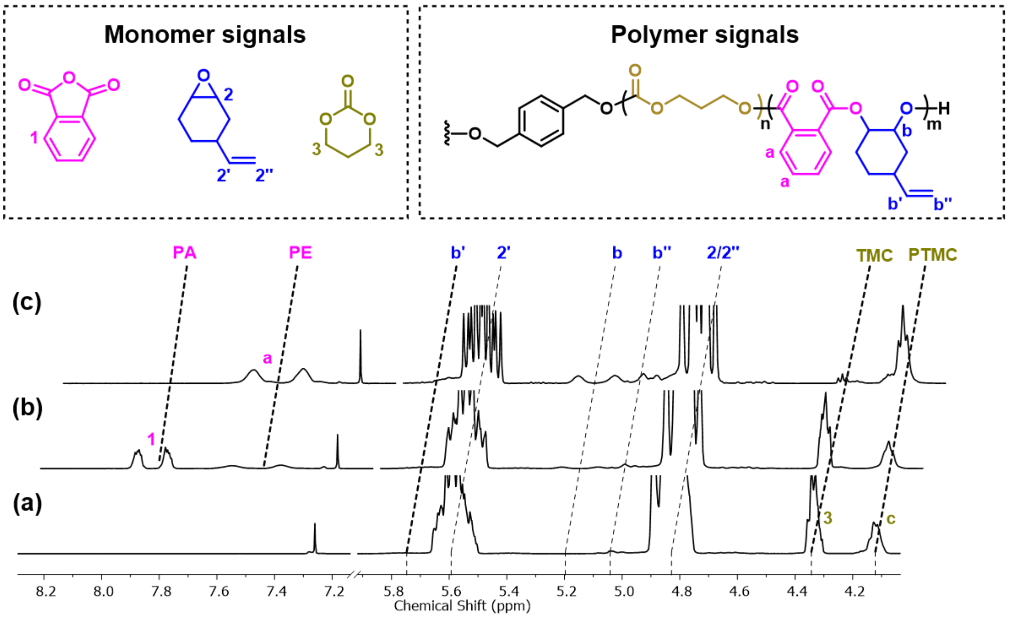

Figure S2. 'Switching' between TMC ROP and PA/vCHO ROCOP. (a) ^{1}H NMR ($CDCl_3$) after 20 min at RT showing 30% conversion of TMC to PTMC. Polymerization conducted in vCHO solvent, Initial TMC concentration = 1 M, [LZn_2Ph_2]:[BDM]:[TMC] = 1:4:500. (b) Addition of PA/vCHO and 1 h at 100 °C showing conversion of PA to PE(v) with no further PTMC production. (c) After 5 h at 100 °C correlating to full conversion of PA to PE(v). Only after full PA conversion does TMC ROP resume.

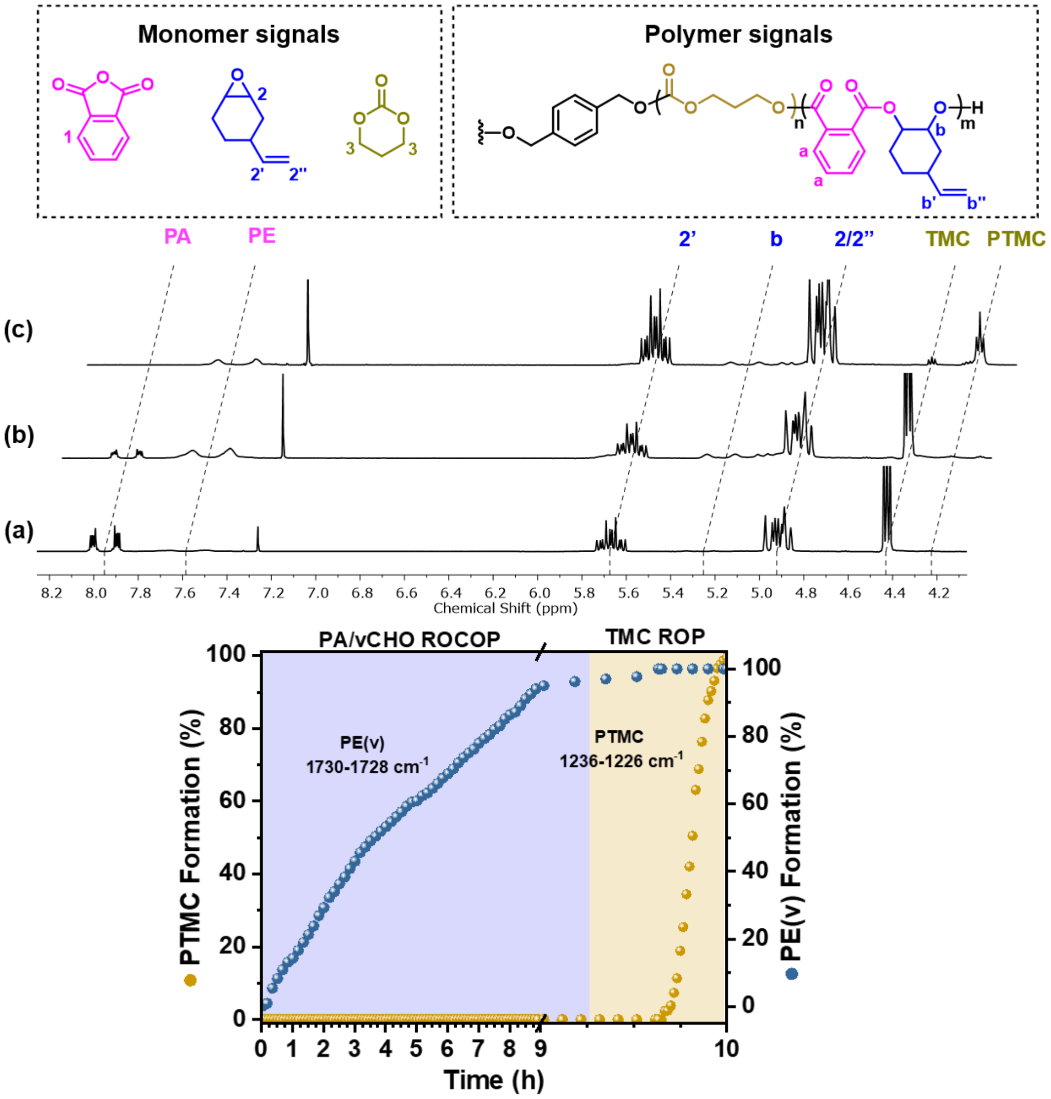

Figure S3. TMC/PA/vCHO one-pot 'switch'. (a) ^{1}H NMR spectra ($CDCl_3$) after 24 h at RT. (b) 24 h at RT then 2 h at 100 °C. (c) 24 h at RT then 4 h at 100 °C. Bottom: *In situ* React IR monitoring of one-pot polymerization at 100 °C. Conversion determined by IR absorbance peaks for PTMC (1236 – 1226 cm^{-1}) and PE(v) (1730-1738 cm^{-1}).

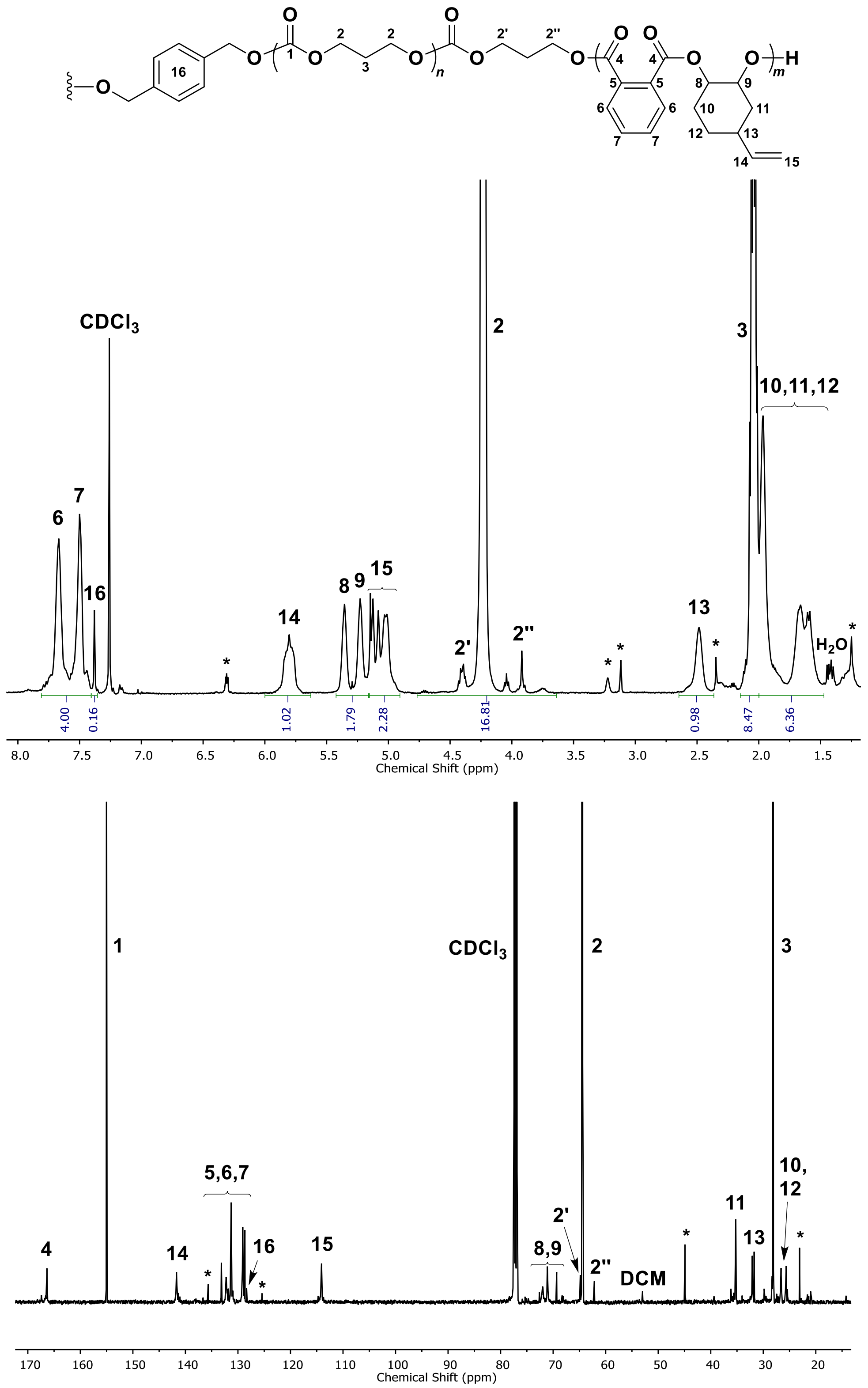

Figure S4. NMR characterization ($CDCl_3$) of PE(v)-b-PTMC-b-PE(v). Top: ^{1}H NMR spectrum of triblock polymer with overall $M_{n,SEC}$ = 17 kg mol^{-1} and 39 wt % PE(v) by NMR integration. Bottom: Corrosponding $^{13}C\{^{1}H\}$ spectrum. At these lower M_n, junctions units between blocks can be observed (2',2''). * = residual vCHO.

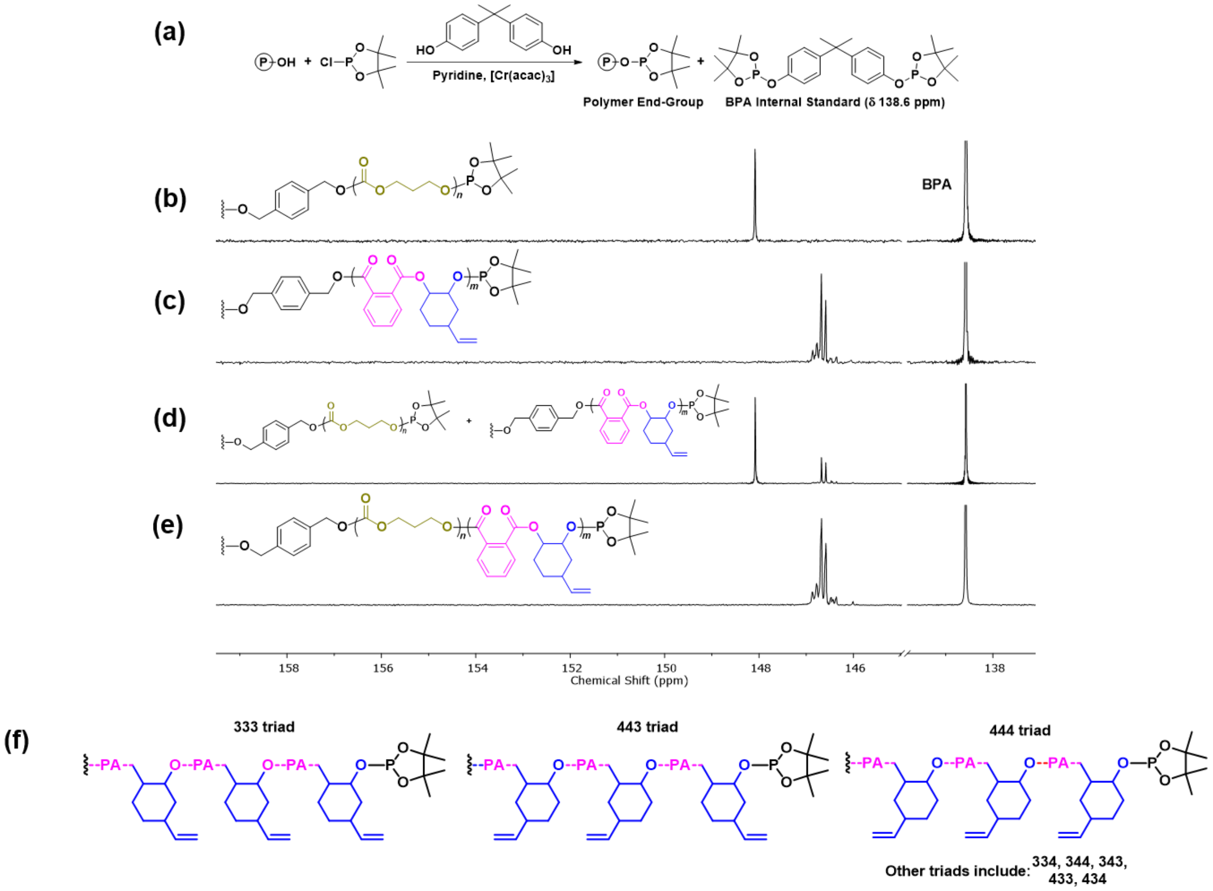

$$\text{number of regioisomers}^{\text{influence range}} = \text{number of signals}: \quad n^i = s \iff i = \log_n(s) = \log_2(8) = 3$$

Figure S5. Polymer chain end-group analysis. (a) Reaction of polymer hydroxyl chain ends with phosphorus reagent (see experimental details above) for $^{31}P\{^{1}H\}$ spectroscopy ($CDCl_3$) (b) – (d). All synthesized triblock polymers PE(v)-PTMC-PE(v) showed only PE(v) end-groups at 146.9-146.4 ppm. Shown for comparison are PTMC (148.1 ppm) and PE(v) pure polymers (147-146.4 ppm) and a 50:50 blend of PTMC and PE(v) (148.1 + 147-146.4 ppm). All spectra are referenced to bisphenol A (BPA) internal standard at 138.6 ppm. (f) The multiplicity of the PE(v) end-group is attributed to the possible triads for the PE(v) end-group. Numbers specify the 1,3- and 1,4-regioisomers relative to the end-group.

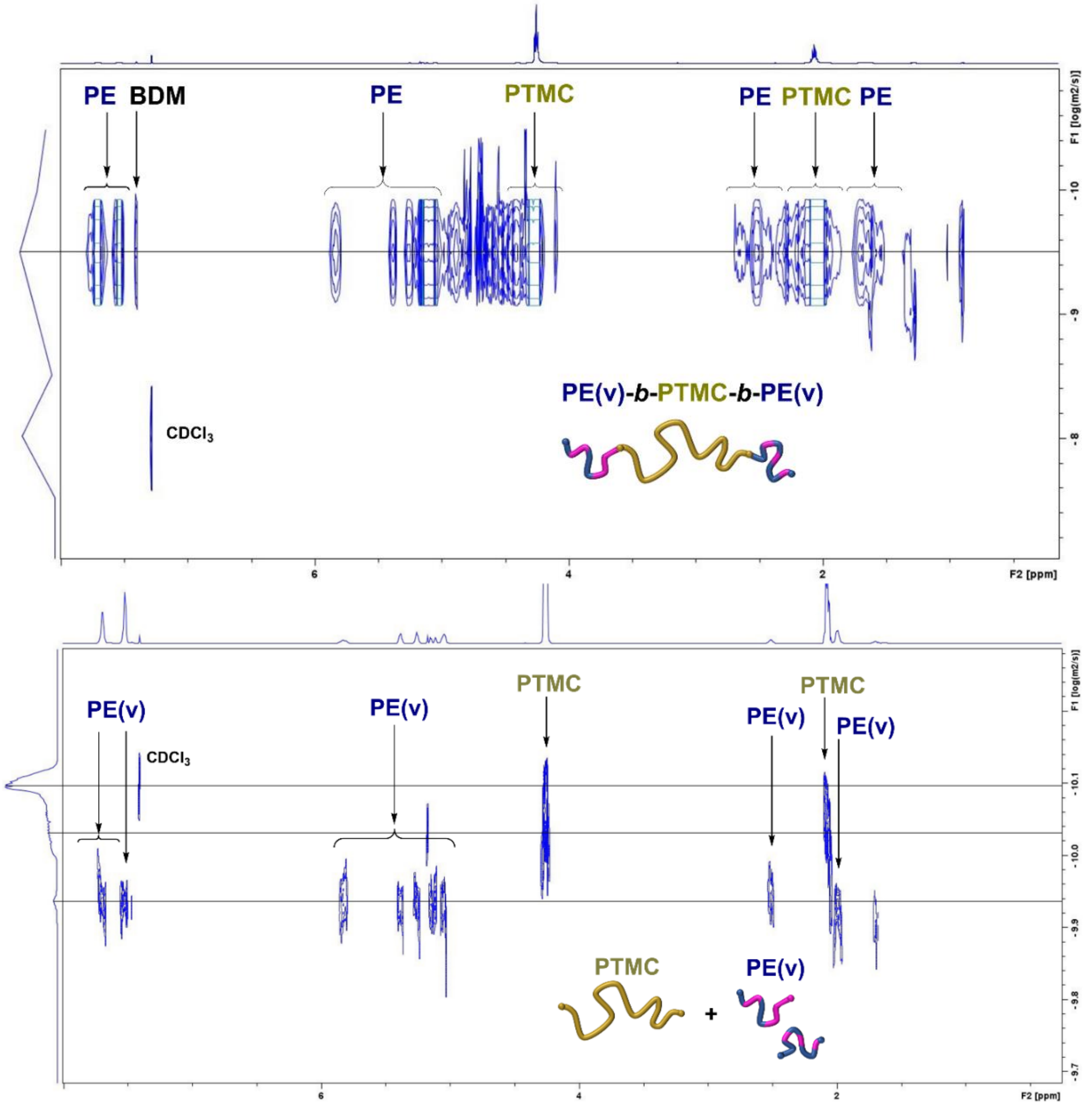

Figure S6. DOSY NMR spectra. Top: PE(v)-*b*-PTMC-*b*-PE(v) triblock copolymer. Bottom: blend of PTMC and PE(v) of similar mass.

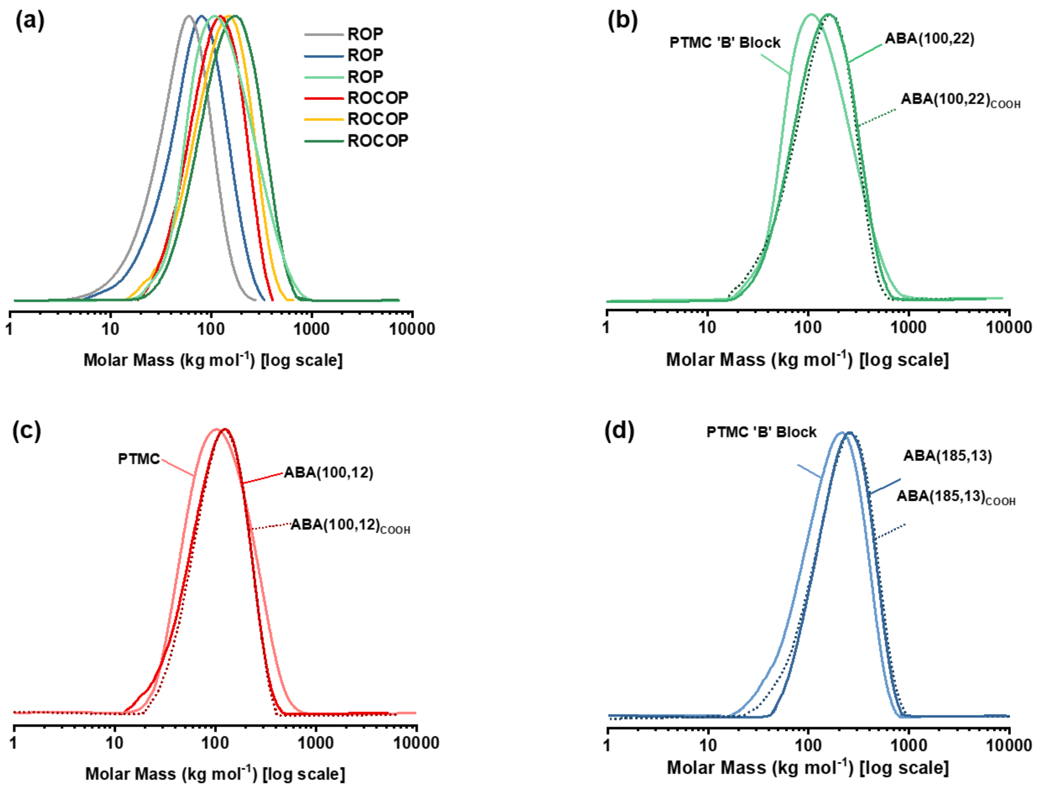

Figure S7. Size Exclusion Chromatography (SEC) of P1-P3. (a) SEC taken of aliquots throughout the synthesis of **P1**. (b)-(c) PTMC SEC was taken as an aliquot of the reaction mixture prior to PA/vCHO addition.

Table S2. Polymerization details for ABA Triblock Synthesis.[a]

Polymer[b]	Cat/BDM/TMC /PA	t_{ROP} (h)	t_{ROCOP} (h)	Conv. TMC (%)[c]	Conv. PA (%)[d]	$M_{n,theo}$ (kg mol^{-1})[e]	$M_{n,NMR}$ (kg mol^{-1}) [f]	$M_{n,SEC}$ (kg mol^{-1}) [$Đ$][g]
ABA(12,26)	1/4/700/125	0.5	1.0	70	70	18	11.7	11.8 [1.56]
ABA(13,21)	1/4/800/100	0.7	1.5	70	96	21	12.9	13.4 [1.65]
ABA(17,39)	1/4/500/250	0.2	1.5	70	61	19	17.0	17.7 [1.34]
ABA(67,16)	1/2/1600/150	3.0	12	80	>99	77	69	67.0 [1.61]
ABA(99,15)	1/2/1600/150	2.5	12	91	83	91	n.d	99.0 [1.62]
ABA(100,22)	1/4/4000/600	1.0	12	70	95	110	n.d	100 [1.58]
ABA(100,12)	1/4/4000/400	1.0	12	78	96	106	n.d	100 [1.59]
ABA(185,13)	1/2/4000/400	4.0	12	72	98	200	n.d	185 [1.39]

[a] Catalyst = [LZn_2Ph_2]; Initiator/CTA= 1,4-BDM, $[TMC]_0$ = 1.0 M in vCHO (entries 1-3) or THF (entries 4-7). For latter, vCHO is present in excess (1.5 × PA equiv.); ROP was conducted at RT and ROCOP at 80 °C. [b] ABA(x, y), x = overall $M_{n,SEC}$ and y = wt% PE(v). [c] Determined by ^{1}H NMR integrals of TMC (δ 4.45 ppm) and PTMC (δ 4.23 ppm). TMC conversion remained unchanged after the ROCOP step. [d] Conversion determined by ^{1}H NMR integrals of PA (δ 8.05-7.80 ppm) and PA in PE(v) (δ 7.85-7.40 ppm). [e] Theoretical overall molar mass calculated from initial monomer-to-initiator ratio, monomer conversion from NMR and molecular weights for PTMC and PE(v) repeat units of 102.1 g mol^{-1} and PE(v)= 272.2 g mol^{-1}, respectively. [f] M_n from ^{1}H NMR integrals: BDM (δ 7.38 ppm, 4H), PTMC (δ 4.23 ppm, 4H) and PE(v) (δ 7.85-7.40 ppm, 4H). [g] $M_{n,\,SEC}$ and $Đ$ determined by SEC ($CHCl_3$ eluent, RI detector) vs narrow PS standards. $Đ = M_w/M_n$. For all isolated polymers, end-group titration confirmed only the ROCOP block as the end-blocks.

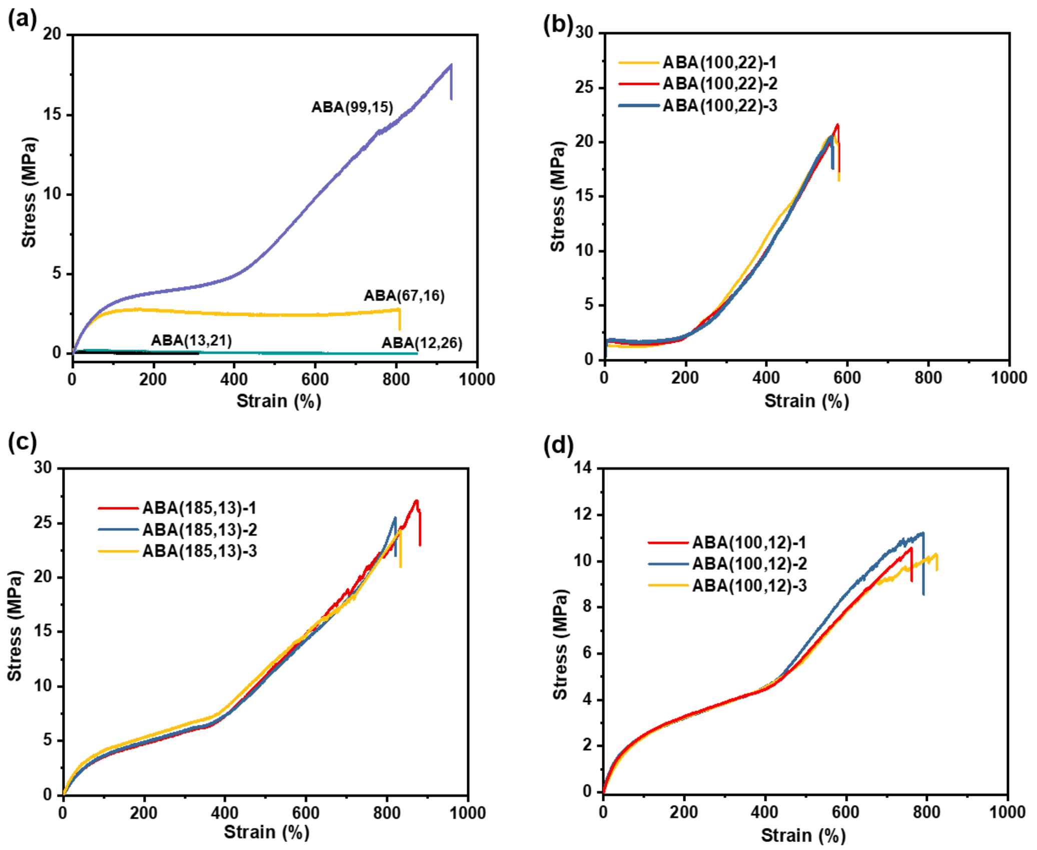

Figure S8. Additional stress-strain curves for PE(v)-PTMC-PE(v) triblock polymers. (a) Influence of overall polymer M_n on mechanical properties (curves are representative of at least three repeats). (b)-(d) Repeats for ABA triblock polymers: **P1** = ABA(100,22), **P2** = ABA(100,12) and **P3** = ABA(185, 13).

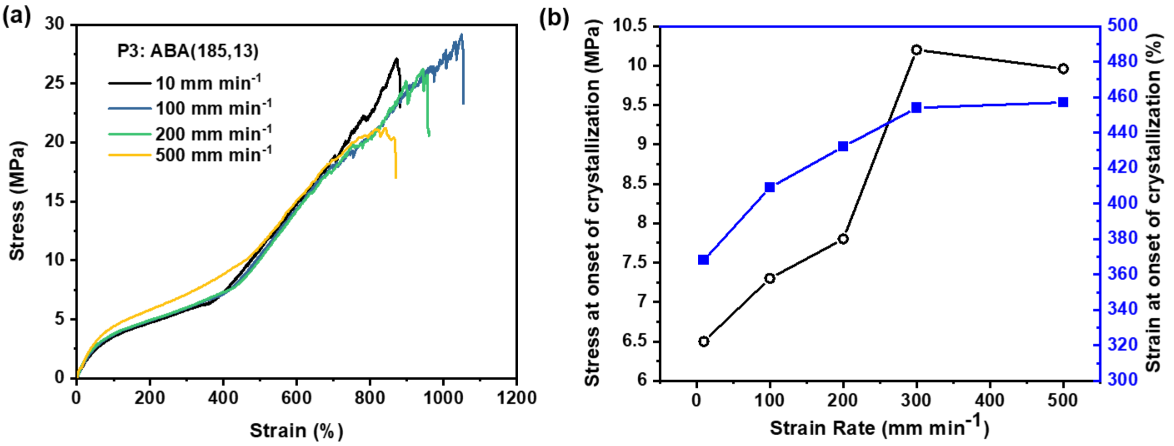

Figure S9. Strain rate dependence of stress-strain behaviour for P3. (a) Stress-stress curves measured at strain rates from 10-500 mm min^{-1}. (b) Dependence of the onset of strain-induced crystallization with strain rate.

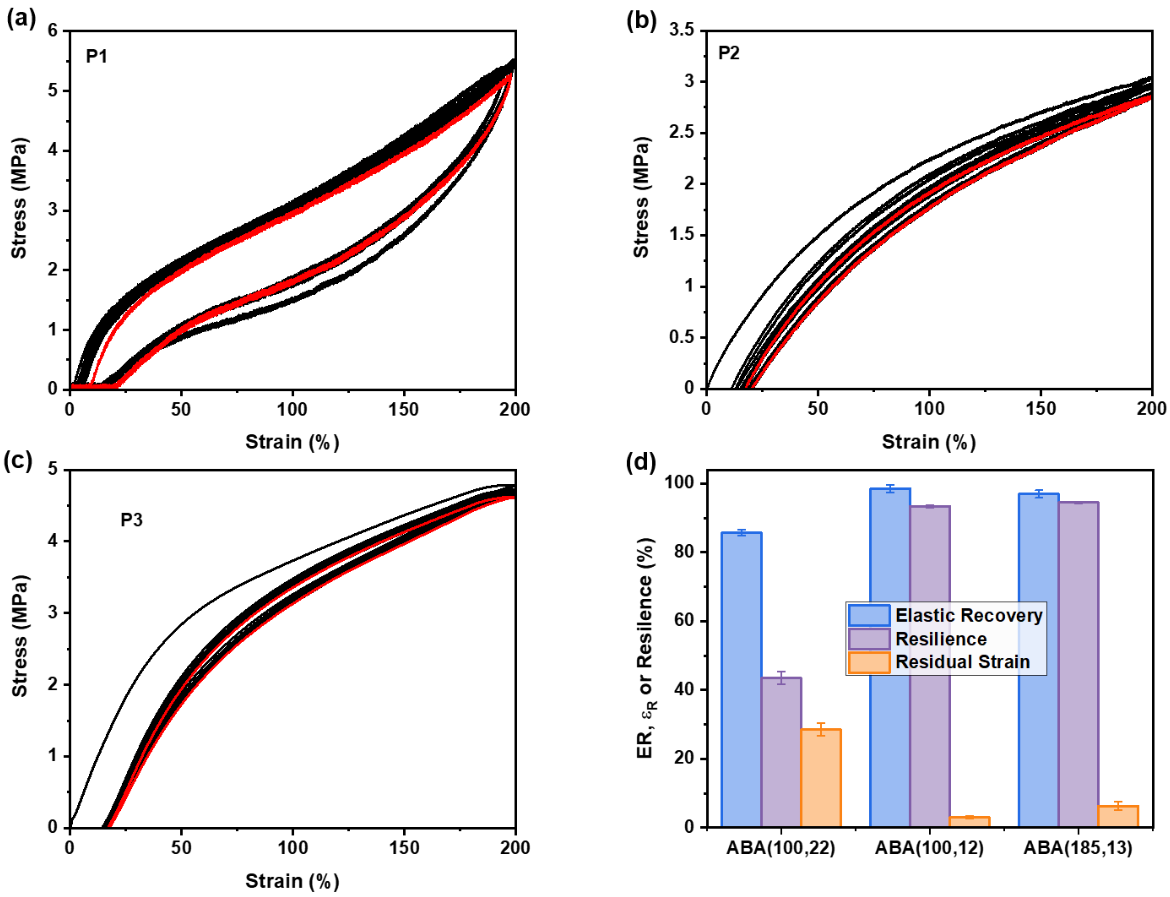

Figure S10. Cyclic tensile testing of ABA polymers to 200% strain. 10 cycles shown for (a) **P1**/ABA(100,22) after pre-processing by stretching to 100% strain: Elastic recovery (ER) = 85.7 ± 0.9 %, residual strain (ε_R) = 28.5 ± 1.8 %, resilience = 43.5 ± 1.8 %. (b) **P2**/ABA(100,12). ER = 98.5 ± 1.2 %, ε_R = 3.0 ± 0.4%, resilience = 93.4 ± 0.4 %. (c) **P3**/ABA(185,13): ER = 96.9 ± 1.1 %, ε_R = 6.3 ± 1.3 %, resilience = 94.5 ± 0.3 %. (d) Bar chart summarizing elastic recovery, residual strain and resilience.

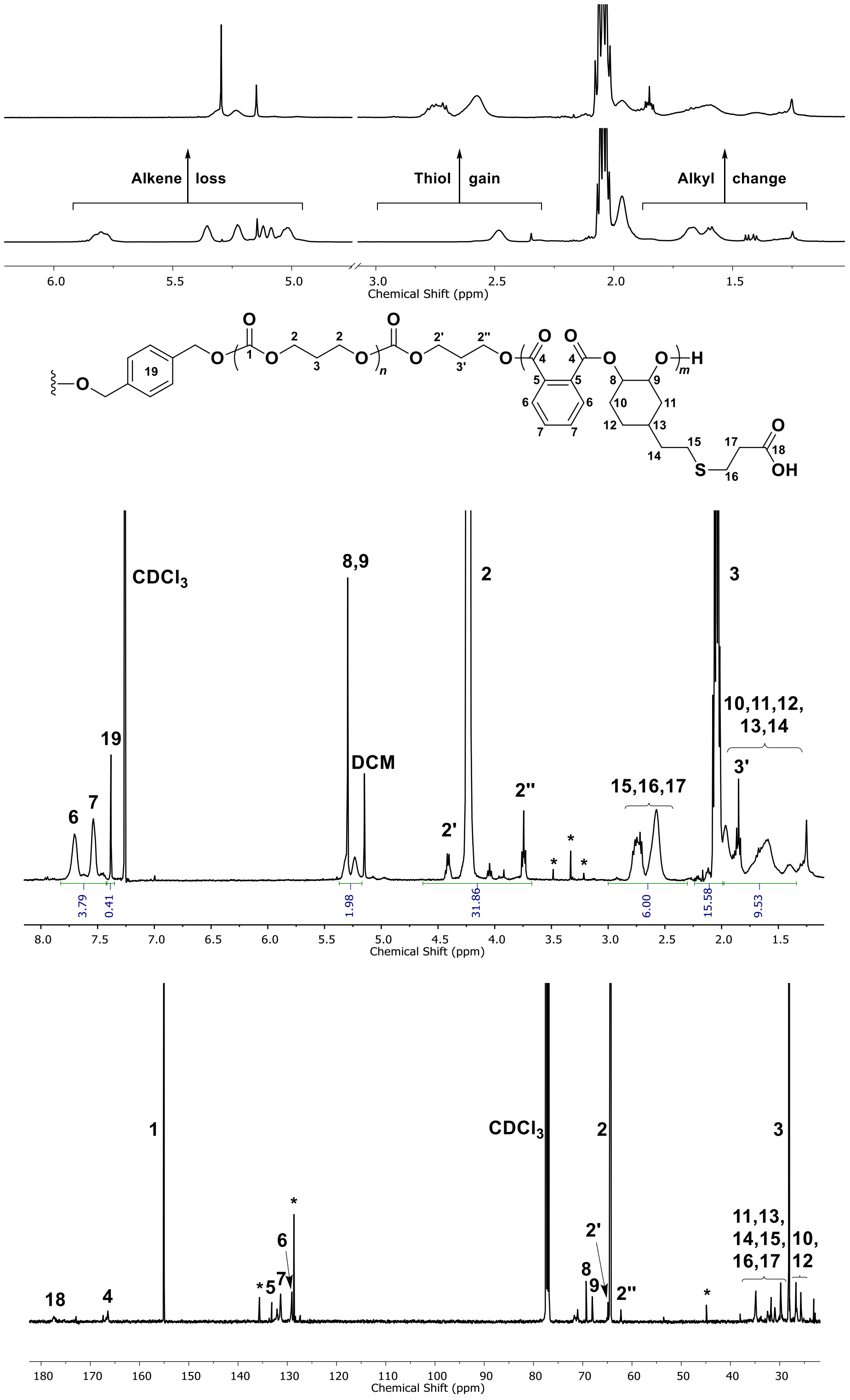

Figure S11. NMR characterization ($CDCl_3$) of COOH modified ABA polymer. Top: ^{1}H NMR showing differences before and after thiol-ene reaction. Middle: ^{1}H NMR spectrum of ABA(17,39)$_{COOH}$. Bottom: Corrosponding $^{13}C\{^{1}H\}$ NMR spectra. * = trace DMPA (thiol-ene UV initiator). 2’ and 2’’ are assigned to polymer junction units. No transcarbonation of PTMC is observed based on C=O at 154 ppm.

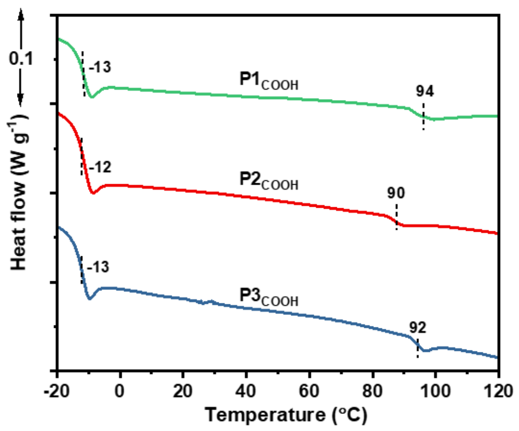

Figure S12. DSC traces of ABA$_{COOH}$. All three functionalized polymers show a slight decrease in the upper T_g (90-94 °C) compared to **P1-P3**. This is attributed to the increased degrees of freedom for the thio-ether carboxylic acid substituents compared with vinyl groups. This rationale is further supported by the T_g decrease being most significant for **P1$_{COOH}$** and **P3$_{COOH}$**, which both feature higher polyester degrees of polymerization (DP = 41-44) compared to **P2$_{COOH}$** (DP = 22).

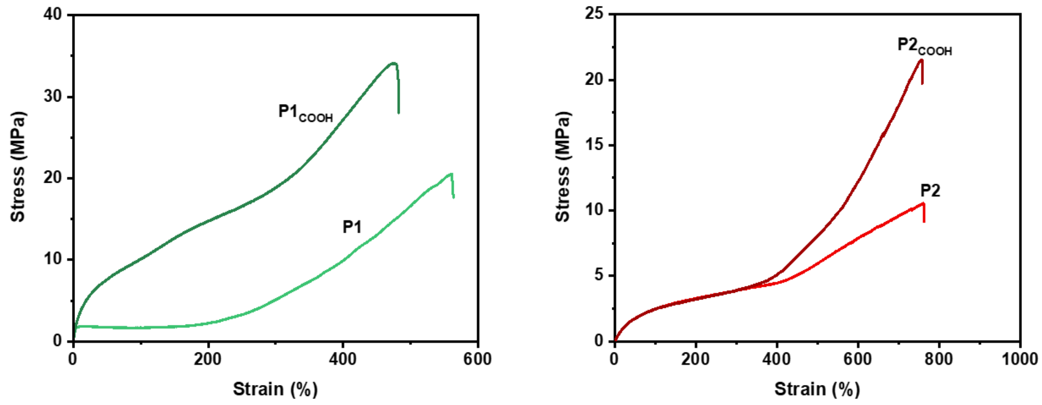

Figure S13. Additional stress-strain curves comparing unmodified P1-P2 and COOH-functionalized P1-P2$_{COOH}$. Experiments were conducted under tension at 10 mm min^{-1}. Representative curves of at least three measurements are shown (see below).

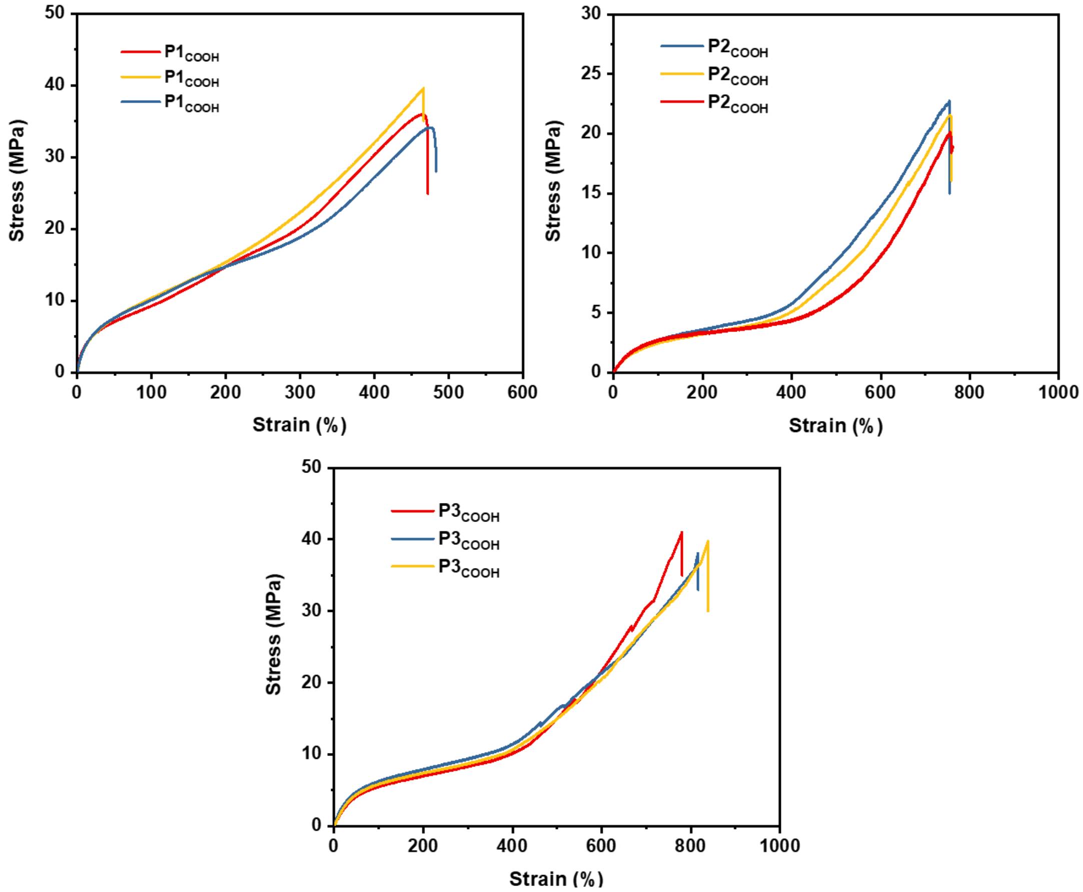

Figure S14. Uniaxial tensile testing data for P1-P3$_{COOH}$. Stress-strain curves showing 3 repeats of dumb-bells specimens as labelled. Strain rate = 10 mm min^{-1}.

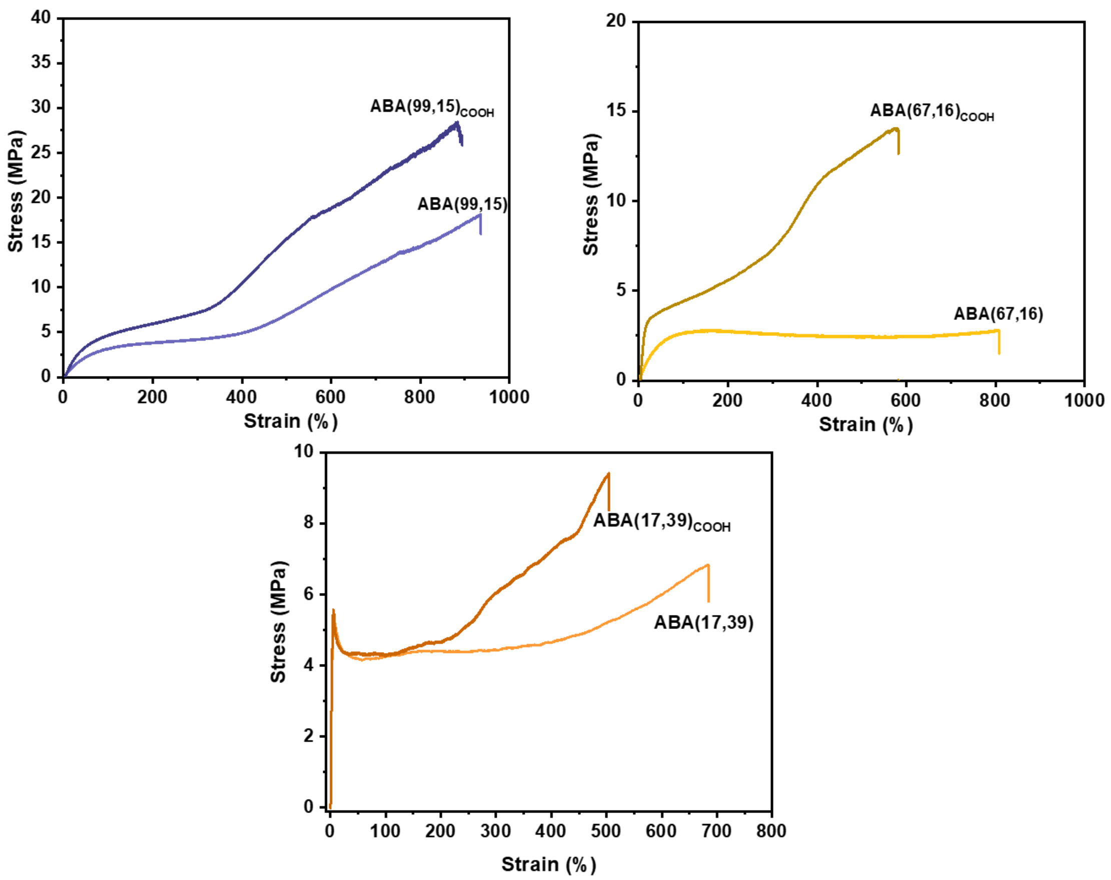

Figure S15. Development of strain-induced crystallization behaviour on carboxylic acid functionalization. Exemplar stress-strain curves for uniaxial extension measurements of ABA triblock copolymers and modified ABA triblock copolymers.

Table S3. Additional mechanical data for ABA and ABA$_{COOH}$.[a]

Sample[b]	E_y (MPa)	σ_b(MPa)	ε_b (%)	ER (%) @ 200%
ABA(12,26)	6.4 ± 1.0	n.d	950 ± 100	n.d
ABA(12,26)$_{COOH}$	8.8 ± 0.6	n.d	590 ± 35	84 ± 12
ABA(13,21)	2.0 ± 0.8	n.d	355 ± 45	n.d
ABA(13,21)$_{COOH}$	0.8 ± 0.1	n.d	180 ± 20	n.d.
ABA(17,39)*	139 ± 12	7.5 ± 0.6	715 ± 31	71 ± 5.2
ABA(17,39)$_{COOH}$*	175 ± 15	10 ± 0.6	500 ± 23	69 ± 2.5
ABA(67,16)	2.8 ± 0.5	2.3 ± 0.4	786 ± 30	67 ± 5
ABA(67,16)$_{COOH}$	24 ± 1.0	19.5 ± 2.6	745 ± 39	88 ± 3
ABA(99,15)	6.1 ± 0.2	20.9 ± 8.8	1034 ± 45	81 ± 4
ABA(99,15)$_{COOH}$	10.9 ± 0.4	38.3 ± 2.6	847 ± 93	90 ± 2

[a] Determined by uniaxial tensile testing at 10 mm min^{-1} strain rate: E_y = Young's Modulus (measured in 0.025 - 0.25% strain region), ε_b = stress at break, σ_b = strain at break. ER = Elastic recovery; determined by cyclic tensile testing (10 mm min^{-1}, 10 cycles). All values are reported as mean values ± std of at least three measurements. n.d = Not determined because the material was too weak. [b] ABA(x,y) refers to a triblock polymer with overall $M_{n,SEC}$ of 'x' kg mol^{-1} and 'y' wt% A block. Subscript 'COOH' refers to functionalizing all vinyl groups with mercaptopropionic acid (MPA). * defined yield point at: 5.1 ± 0.1 MPa and 7.6 ± 0.5 % for ABA(17,39); 5.9 ± 0.3 MPa and 5.1 ± 0.4 % for ABA(17,39)$_{COOH}$.

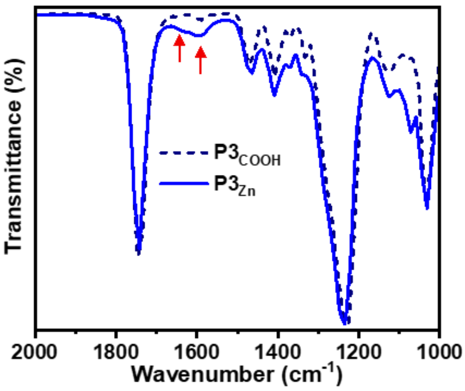

Figure S16. FTIR spectroscopic analysis of triblock polymer films. The arrows indicate the asymmetric and symmetric Zn(II)-carboxylate stretches in **P3$_{Zn}$** consisted with the targeted 10 % Zn functionalization. Analysis of **P3$_{Zn}$** by NMR spectroscopy was limited by its solubility.

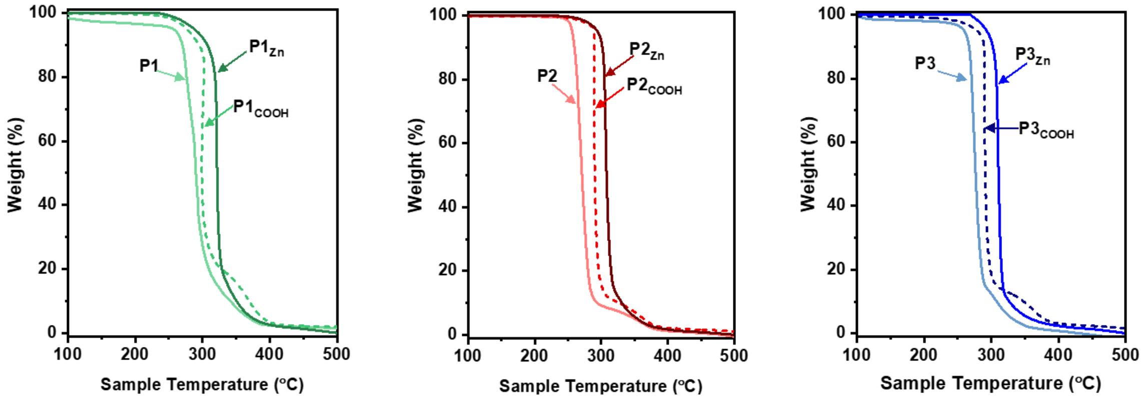

Figure S17. TGA thermograms.

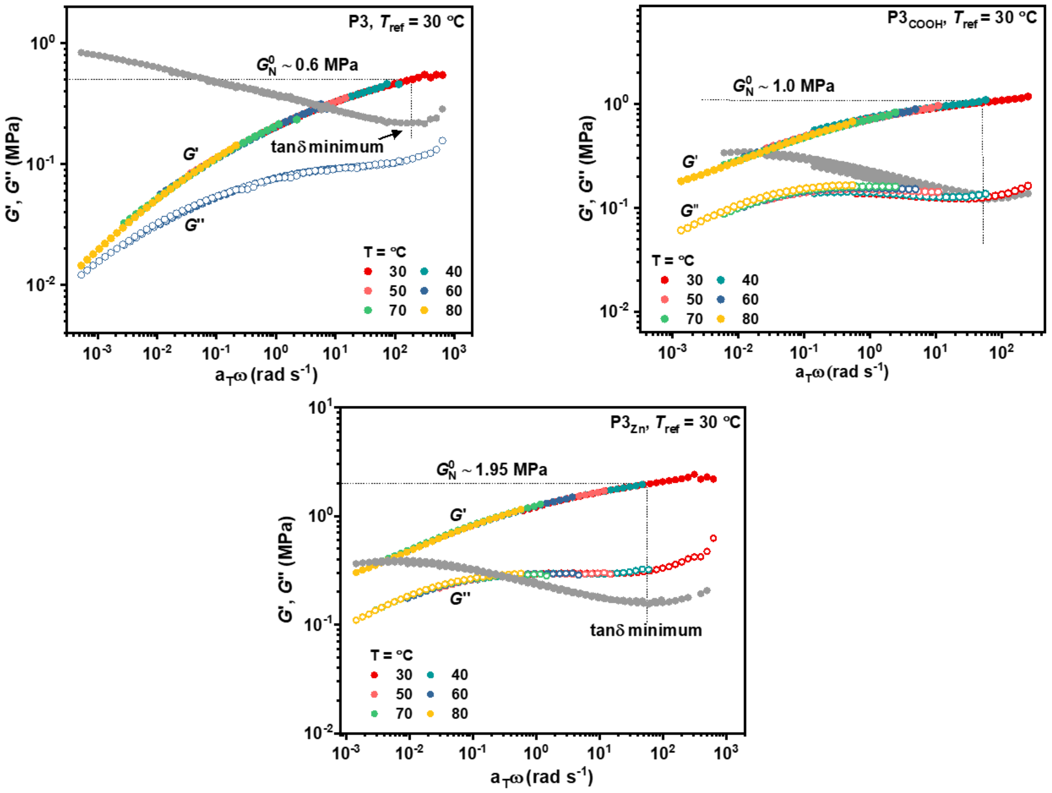

Figure S18. Time-temperature superposition (TTS) curves. Frequency sweeps conducted at T = 30, 40, 50, 60, 70 and 80 °C in the linear viscoelastic region (1 % amplitude strain were used to generate TTS curves at a reference temperature of 30 °C. For **P3** WLF fit: c1 = 35.3, c2 = 479 K (R^2 = 0.99). For **P3$_{COOH}$** WLF fit: c1 = 10, c2 = 196 K (R^2 = 0.992). For **P3$_{Zn}$** WLF fit: c1 = 9.6, c2 = 126 K (R^2 = 0.999). G_N^0 was estimated from the *G*' curve at the minimum in tanδ as described elsewhere.[5]

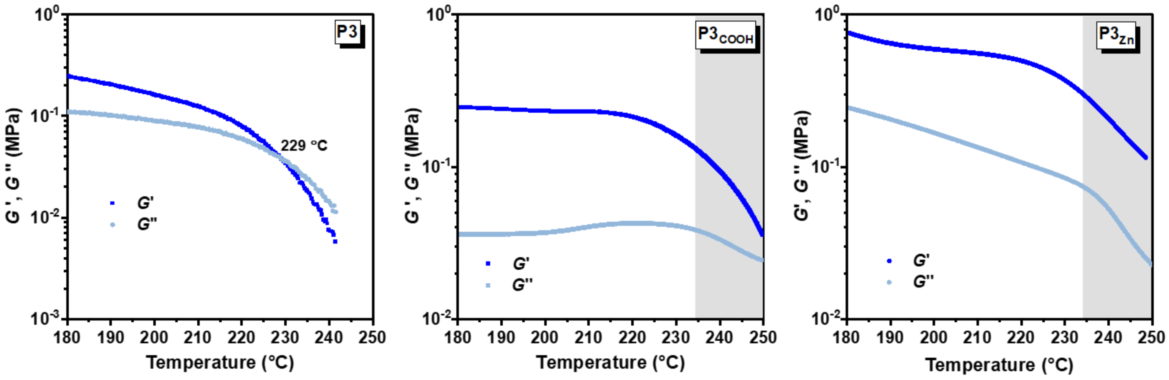

Figure S19. Additional rheology data. *G*' and *G*'' vs T for **P3** series. All measurements were conducted in the linear viscoelastic region: 1% amplitude stain, 1 Hz frequency. 2 °C min^{-1} heat rate. For **P3**, G' to G'' crossover occurred at 229 °C. For **P3$_{Zn}$**, the drop in G' onset occurred at ca. 227 °C, but no *G*'-*G*'' crossover is observed up to the 250 °C temperature measured. For **P3$_{COOH}$**, the onset of the drop in *G*' occurred at ca. 217 °C, with *G*'-*G*'' crossover being observed up to the 250 °C.

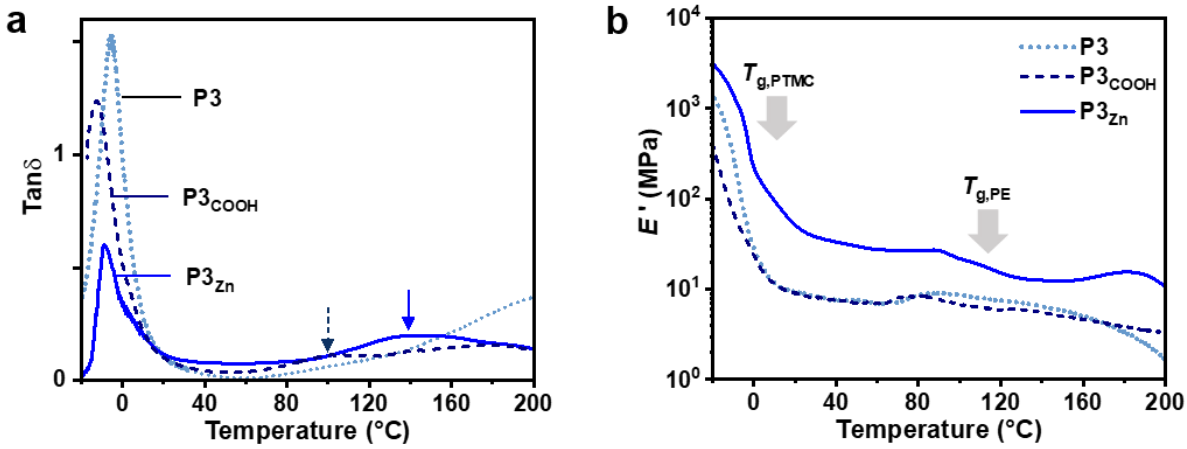

Figure S20. Dynamic mechanically analysis (DMTA) comparing P3, **P3$_{COOH}$** and **P3$_{Zn}$.** (a) Tanδ (ratio of loss, *E*'' to storage, *E*' moduli) as a function of temperature. Peaks in tanδ at -6 to -13 °C are attributed to PTMC glass transitions and showed reduced intensity with end-block physical crosslinking interactions: **P3** > **P3$_{COOH}$** > **P3$_{Zn}$** suggesting restricted chain mobility. For **P3$_{Zn}$,** a broad transition at ~140 °C is attributed to glass transitions in the polyester phases; this is observed at ~100 °C in **P3$_{COOH}$**. Consistent with classic TPE behavior, there is a plateau in the storage modulus between these upper and lower transitions, and **P3$_{Zn}$** exhibits a higher *E*' plateau than **P3**/**P3$_{COOH}$**. Measurements were conducted at 1% amplitude strain, 1 Hz frequency, 3 °C min^{-1}.

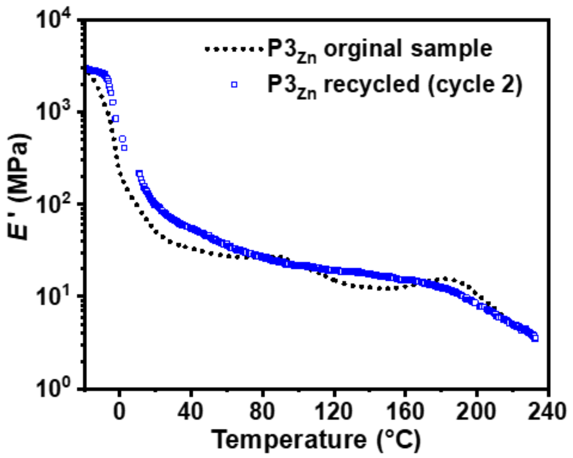

Figure S21. Storage (*E*') moduli as a function of temperature for reprocessed P3$_{Zn}$.

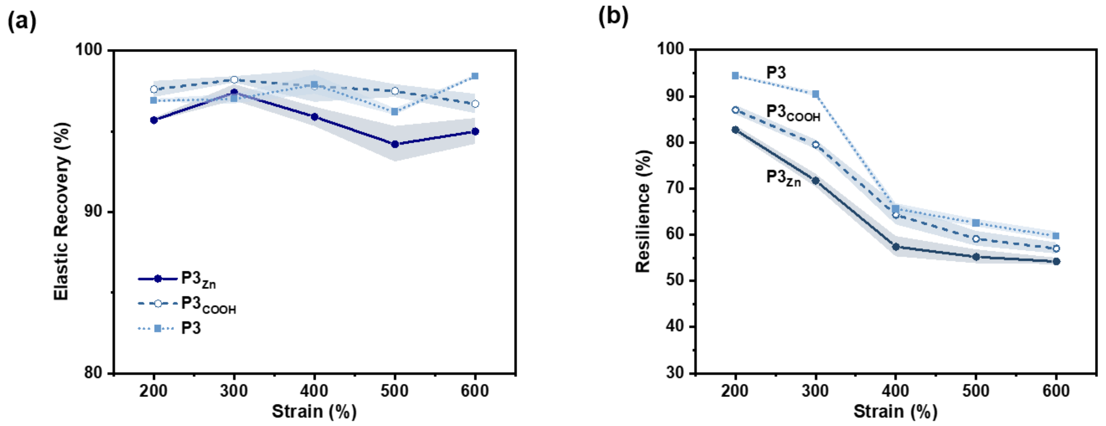

Figure S22. Cyclic tensile testing for $P3_{Zn}$, $P3_{COOH}$ and P3. Shaded regions represent the standard deviation from 10 cycles.

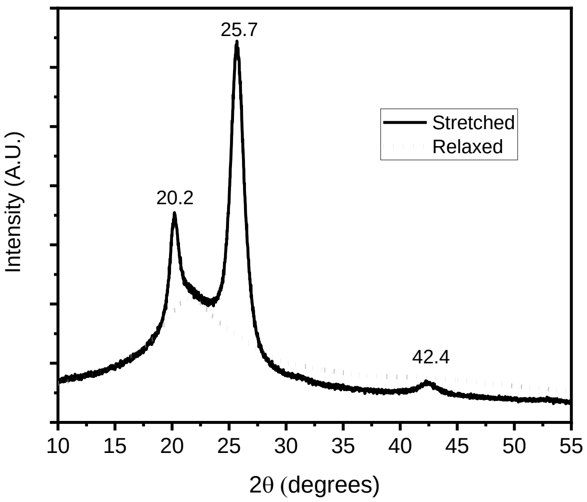

Figure S23. Powder X-ray Diffraction Pattern of $P3_{Zn}$. Percentage crystallinity when stretched = $\chi_c = \frac{I_c}{I_c + I_a}$ where I_c and I_a are crystalline and amorphous regions, respectively.

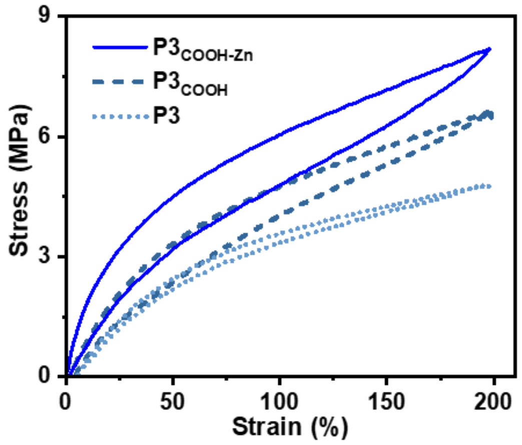

Figure S24 Cyclic tensile tests at 200% strain (only second cycle shown).

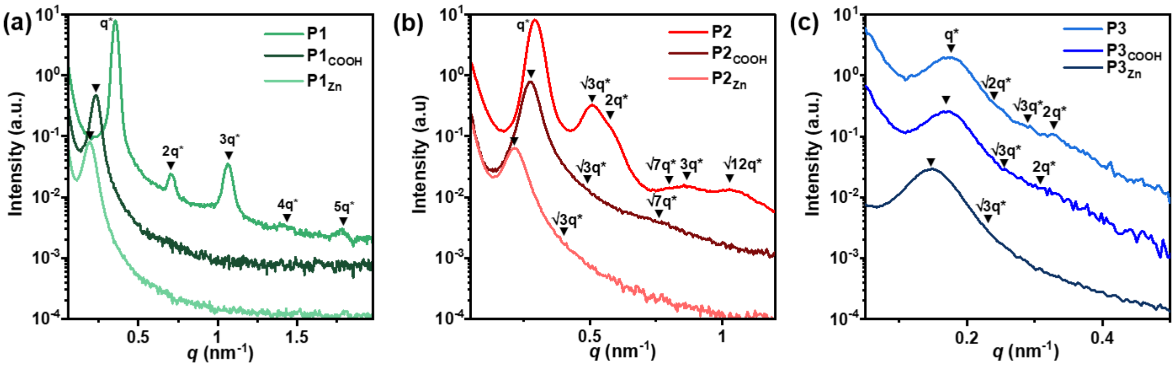

Figure S25. SAXS profiles (RT) for P1-3$_{COOH}$ and P1-3$_{Zn}$. Polymer films were prepared as for mechanical testing. Individual profiles are vertically shifted for clarity. The significant decrease in *q** for **P1** on decoration with COOH (increase in domain size from 17.7 to 27.4 nm) may indicate a change in phase morphology. The shift is less significant for **P2** and **P3**. In all cases, the Zn ionomer results in a shift to lower *q**. The acid functional groups are expected to influence the incompatibility of the blocks ($\chi_{PE\text{-}PTMC}$) and potentially the volume fraction of hard block, both of which could influence the phase separation behaviour.

Table S4. Summary of TPE properties.[a]

Sample	E_y (MPa)	UTS (MPa)	ε_b (%)	ER (%)	U_T (MJ m^{-3})	T_{g1},T_{g2} (°C)[b]	$T_{d,5\%}$ (°C)[c]	*D* (nm)[d]
**P1**	38.7 ± 1.6	20.7 ± 1.0	502 ± 30	85.7 ± 0.9	50 ± 2	-13, 123	268	17.7
**P1**$_{COOH}$	41.6 ± 0.7	36.5 ± 2.8	469 ± 16	84.1 ± 2.0	88 ± 2	-13, 94	300	27.4
**P1**$_{Zn}$	59.7 ± 8.6	54.3 ± 0.4	584 ± 68	n.d	188 ± 16	n.d	319	32.9
**P2**	4.6 ± 0.3	10.7 ± 0.5	791 ± 31	98.5 ± 0.2	43 ± 3	-13, 100	266	21.5
**P2**$_{COOH}$	5.7 ± 0.3	21.0 ± 1.0	755 ± 12	98.2 ± 0.1	56 ± 6	-12, 90	279	22.7
**P2**$_{Zn}$	25.3 ± 2.8	48 ± 2.0	950 ± 36	n.d	170 ± 13	n.d	304	29.3
**P3**	7.1 ± 0.7	25.9 ± 1.1	822 ± 42	96.9 ± 1.1	89 ± 9	-14, 115	269	35.7
**P3**$_{COOH}$	8.6 ± 0.6	39.3 ± 1.0	812 ± 29	97.6 ± 1.5	121 ± 9	-13, 92, [100**]	280	37.3
**P3**$_{Zn}$	41.6 ± 2.5	62.3 ± 1.2	851 ± 23	95.7 ± 2.1	183 ± 8	-16, 94* [140**]	300	42.2

[a] E_y = Young's modulus in 0.025-0.25 % strain region, UTS = ultimate tensile strength, ε_b = elongation at break, ER = elastic recovery at 200% strain. U_T = Tensile toughness determined from the area under stress-strain curve. [b] Glass transition from DSC. * upper T_g from first heating cycle. **determined from the peak in tanδ from DMTA. [c] Temperature at 5% mass loss from TGA. n.d = not determined. [d] Domain spacings from SAXS.

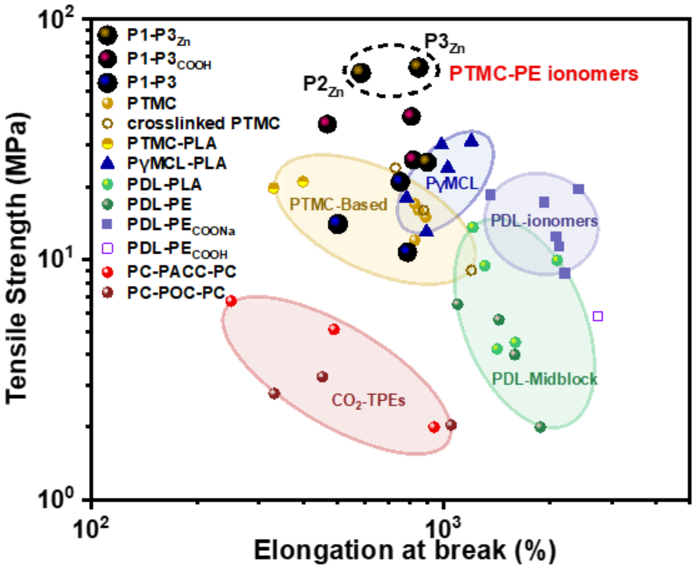

Figure S26. Ashby plot for literature TPEs. P1-P3 (this work); PTMC homopolymers (M_n 114-223 kg mol^{-1});[6] Chemically crosslinked PTMC;[7] PLA-*b*-PTMC-*b*-PLA triblock polymers, where PLA = poly(lactide);[8] Triblock polymers with different rubbery midblocks: PDL = poly(ε-decalactone);[9] PγMCL = Poly(γ-methyl-ε-caprolactone);[10] PE refers to polyesters from PA/CHO ROCOP[9] or PA/vCHO ROCOP; PE_{COONa} refers to ionomers where the carboxylic acid functionality is neutralized with Na; CO_2-TPEs: PC = CO_2/CHO ROCOP polycarbonate; PACC = AGE/CO_2 ROCOP;[11] POC = CO_2/1-octene ROCOP.[12]

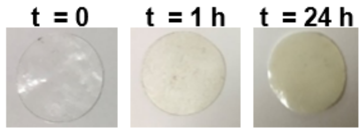

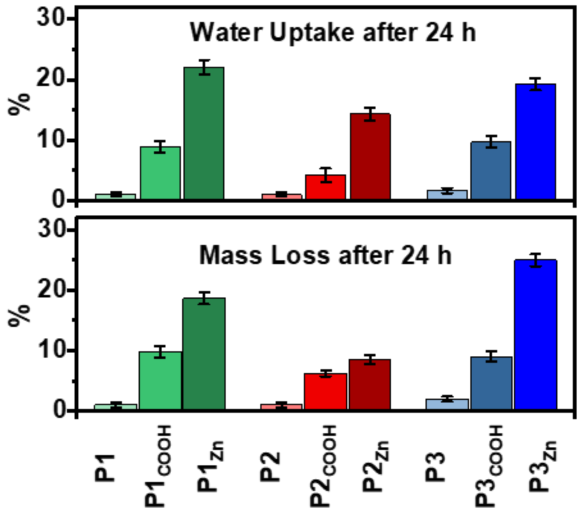

Figure S27. Hydrolytic degradation in aqueous alkaline media. Water uptake and mass loss of polymer discs (0.3 × 16 mm) in water (top) or 1 M $NaOH_{aq}$ (bottom). Picture: discolouration of **P3**$_{Zn}$ in 1M NaOH. Enhancements to the hydrolytic degradability were observed for **P1**$_{Zn}$ and **P3**$_{Zn}$, with higher polyester DP, than **P2**$_{Zn}$, confirming that the faster hydrolysis rates are attributable to the PE component.

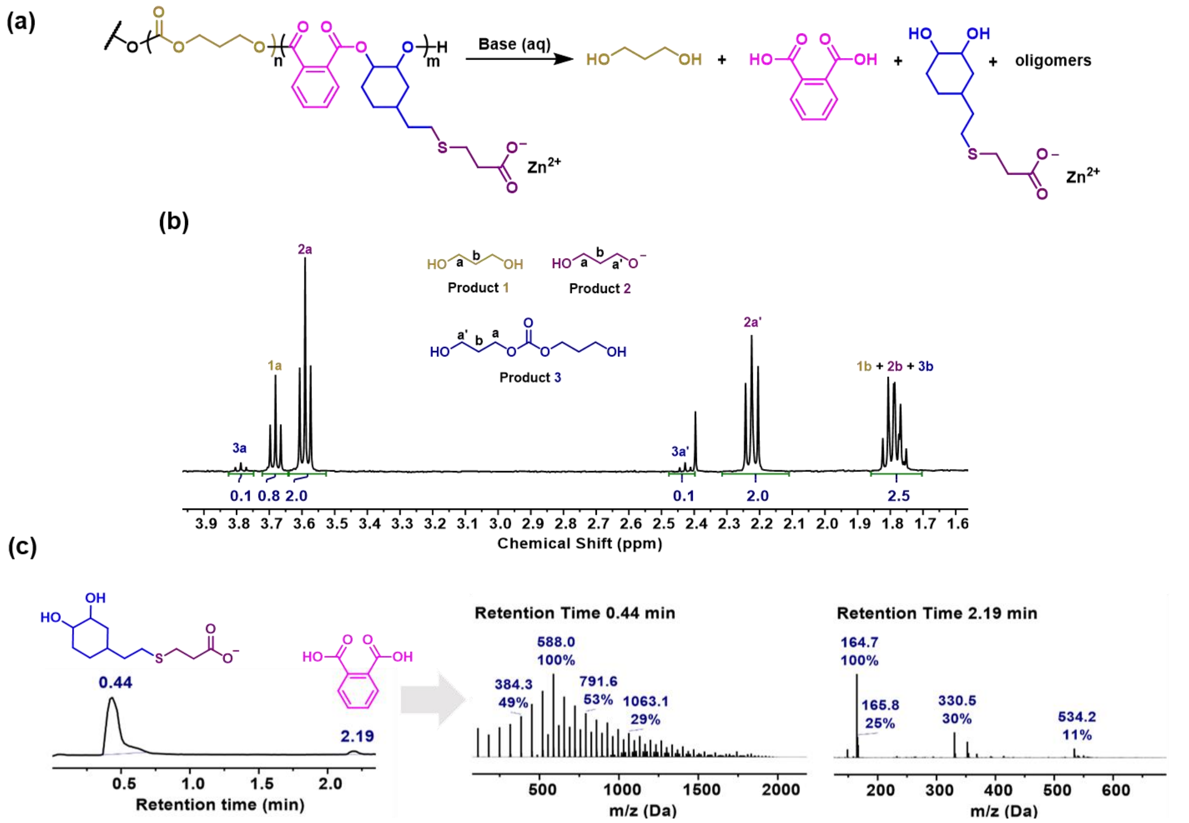

Figure S28. Degradation products of P3$_{Zn}$. (a) Assumed final degradation products. (b) ^{1}H NMR (D_2O) of residue after degradation. (c) LC-MS trace of degradation products. NB. No other peaks were detected at higher retention times. Corresponding mass spectrums of peaks at different retention times. See Table S5 below for the proposed assignment.

Table S5. Proposed Assignment of P3$_{Zn}$ Degradation Products in alkaline media.[a]

Proposed Structure	Proposed Species	Expected *m/z*	Observed *m/z* (relative abundance %)	Error (%)	RT (min)
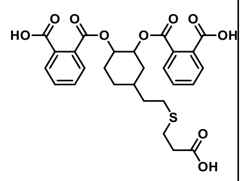	**$[M-3H]^{3-}$**	**180.4**	**180.7 (27)**	-0.17	0.44
	$[M-3H+Na+HCOONa]^-$	316.1	316.4 (41)	-0.09	
	$[2M-6H+3Na]^{3-}$	383.7	384.3 (49)	-0.16	
	**$[M-3H+2Na]^-$**	**587.1**	**588.0 (100)**	-0.14	
	$[M-3H+2Na+HCOONa]^-$	655.8	655.8 (76)	0.00	
	$[M-3H+2Na+2HCOONa]^-$	723.1	723.7 (62)	-0.08	
	$[M-3H+2Na+3HCOONa]^-$	791.1	791.6 (53)	-0.06	
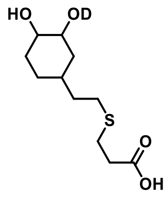	**$[M-H]^-$**	**248.1**	**248.5 (36)**	-0.16	
	$[M-H+HCOONa]^-$	316.0	316.4 (41)	-0.13	
	$[M-H+2HCOONa]^-$	384.1	384.3 (49)	-0.05	
	$[M-H+3HCOONa]^-$	452.1	452.2 (66)	-0.02	
	$[M-H+4HCOONa]^-$	520.1	520.1 (80)	0.00	
	**$[M-H+5HCOONa]^-$**	**588.0**	**588.0 (100)**	0.00	
	$[M-H+6HCOONa]^-$	656.0	655.8 (76)	0.03	
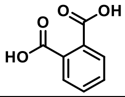	**[M-H]-**	**165.0**	**164.7 (100)**	0.18	2.19
	$[2M-H]^-$	331.0	330.5 (89.7)	0.15	

[a]NMR sample (see above) in D_2O diluted in water for analysis by liquid chromatography-mass spectrometry (LC-MS), mode = negative electron-spray ionization (ESI), formic acid (HCOONa) used to facilitate ionization.

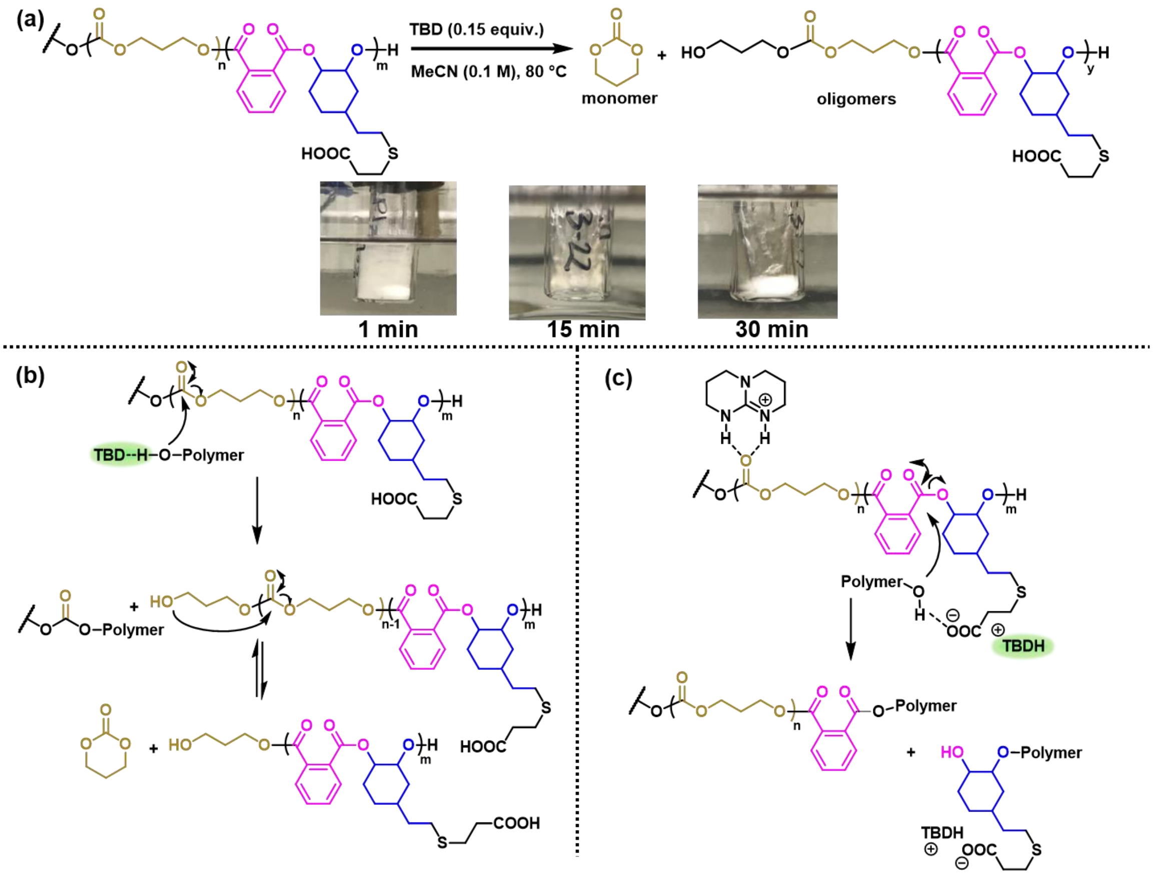

Scheme S2. Proposed reaction scheme for chemical recycling of P1$_{Zn}$. (a) Overall reaction scheme for depolymerizing **P1$_{Zn}$** with TBD. Proposed reaction mechanism: (b) Transcarbonation followed by ring-closing cyclization to TMC monomer. The nucleophile could be either the polymer chain end or added diol. (c) Transesterification of polyester. An acid-base reaction can occur between TBD and pendent COOH on PE, which likely encourages solubilization of the polymer in solvent. TBDH$^+$ and COO$^-$ can activate ester/carbonate C=O and diol, respectively.

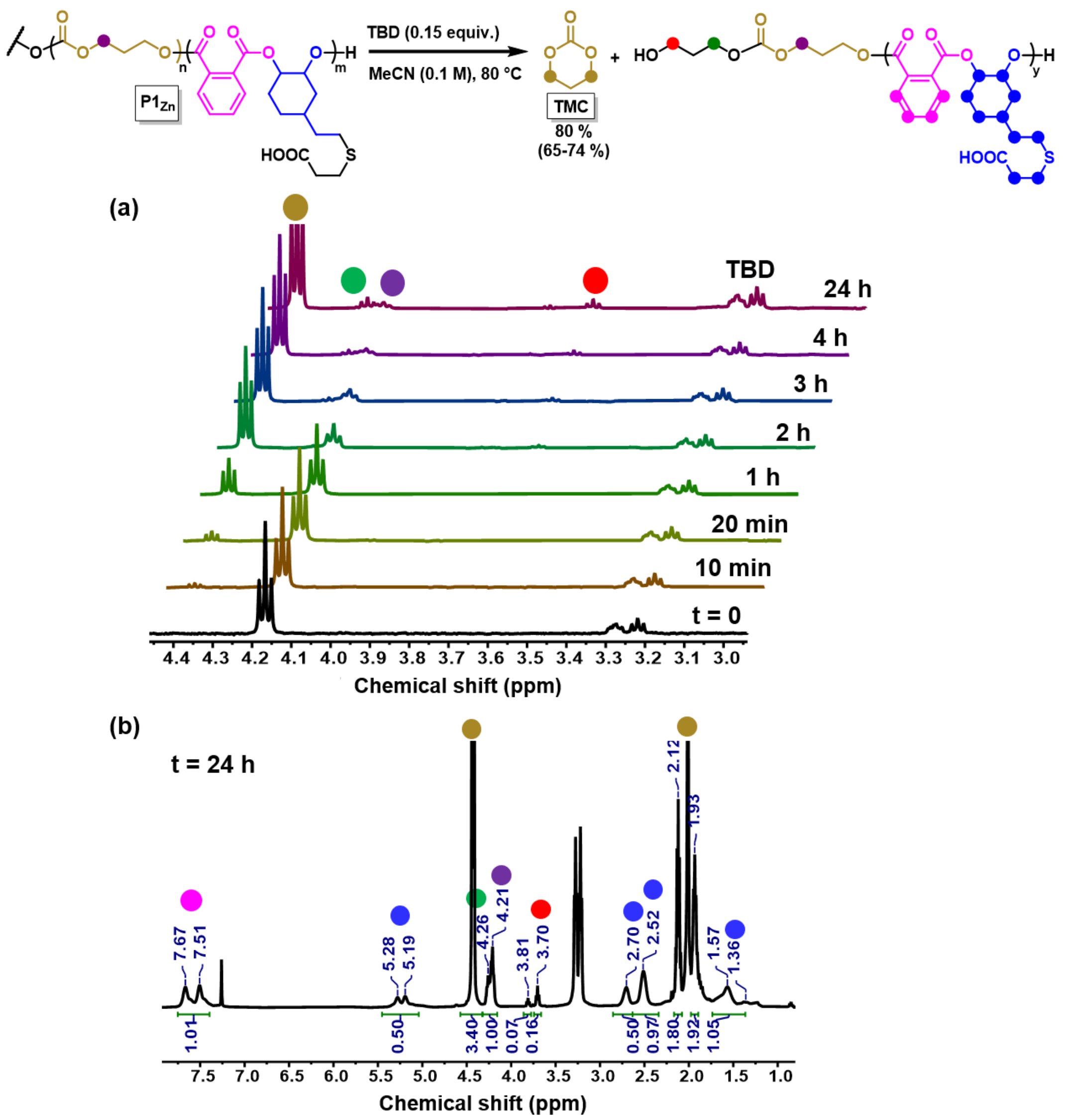

Figure S29. Chemical recycling of $P1_{Zn}$. (a) ^1H NMR ($CDCl_3$) stack of aliquots taken from the reaction mixture and quenched with acetic acid. (b) ^1H NMR ($CDCl_3$) of the crude reaction mixture after 24 hours, quenched with acetic acid and dried under vacuum.

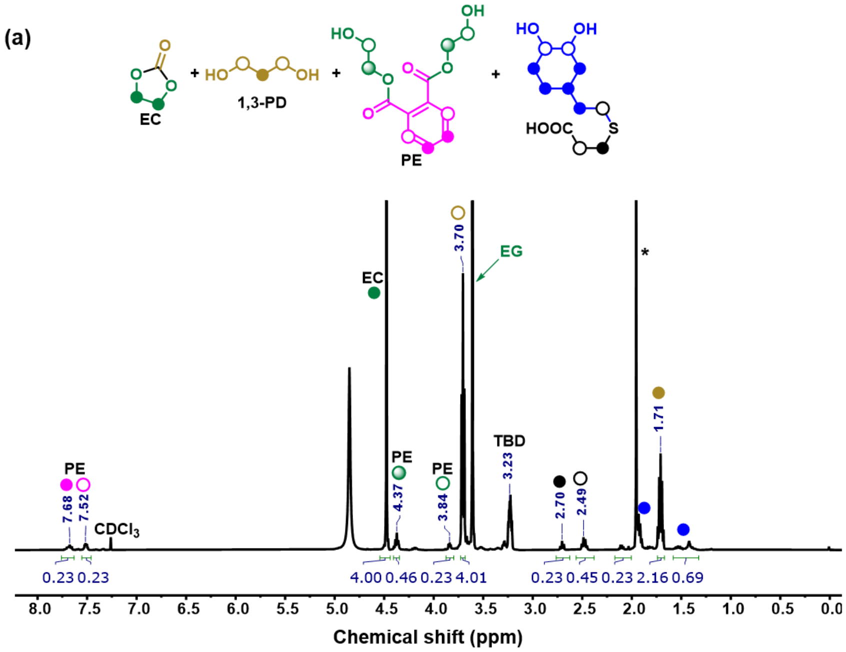
(a)
EC
1,3-PD
PE
HOOC
EC
EG
TBD
CDCl3
Chemical shift (ppm)

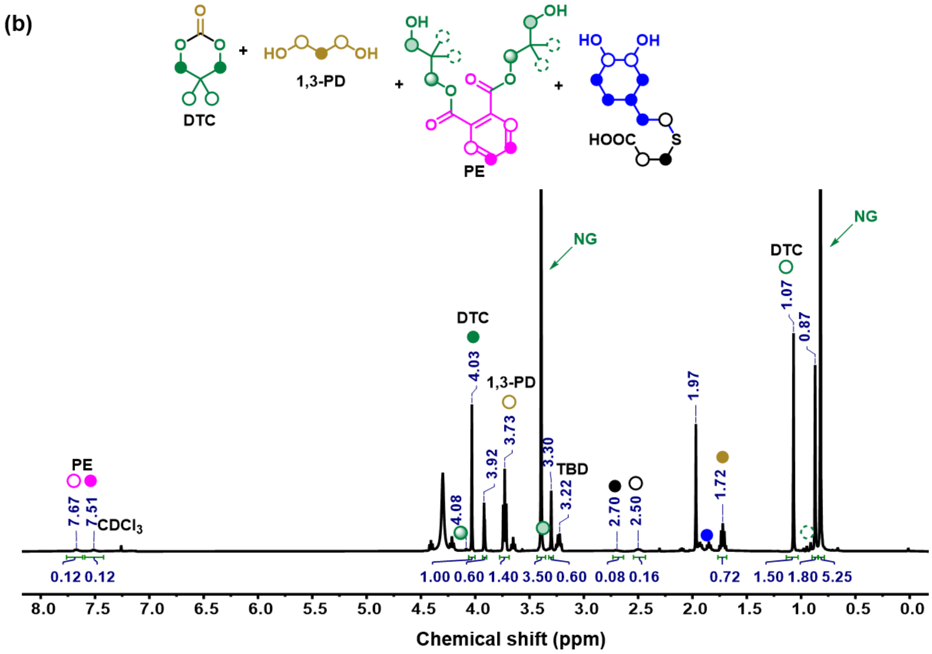
(b)
DTC
1,3-PD
PE
HOOC
NG
TBD
CDCl3
Chemical shift (ppm)

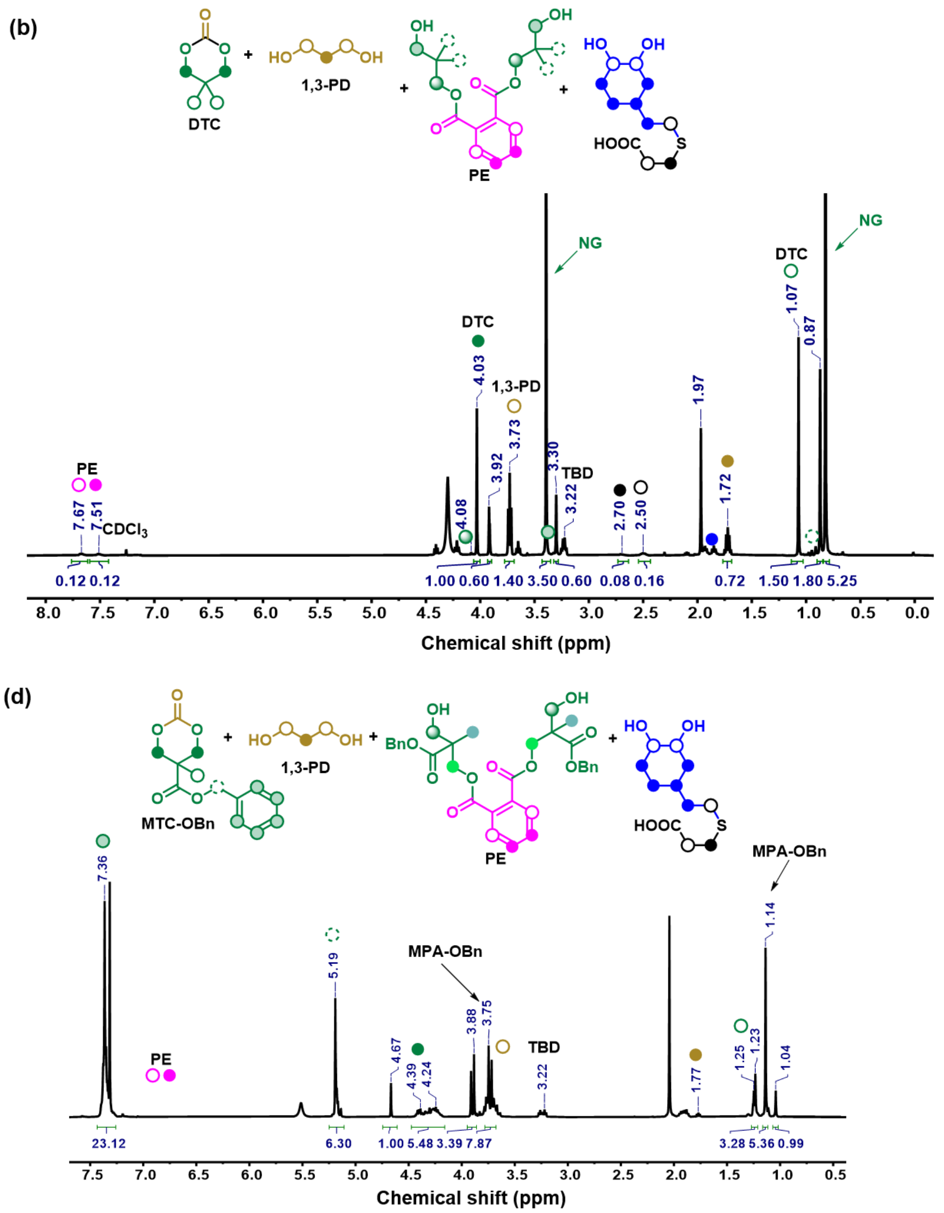

Figure S30. Chemical upcycling of P1$_{Zn}$. ^{1}H NMR ($CDCl_3$) of the crude reaction mixture after quenching with acetic acid (10 equiv. relative to TBD) and removal of the solvent *in vacuo*. In all cases, no polymer was observed by SEC. (a) Diol = ethylene glycol (EG). PE = phthalate diester. (b) Diol = neopentyl glycol (NG). Resonances at 3.92, 3.30 and 0.87 ppm are assigned to linear carbonate side-products. (c) Diol = 2,4-pentanediol. (d) Diol = benzyl 2,2-bis(methylol)propionate (MPA-OBn) synthesized according to literature procedure.[13] * = acetic acid.

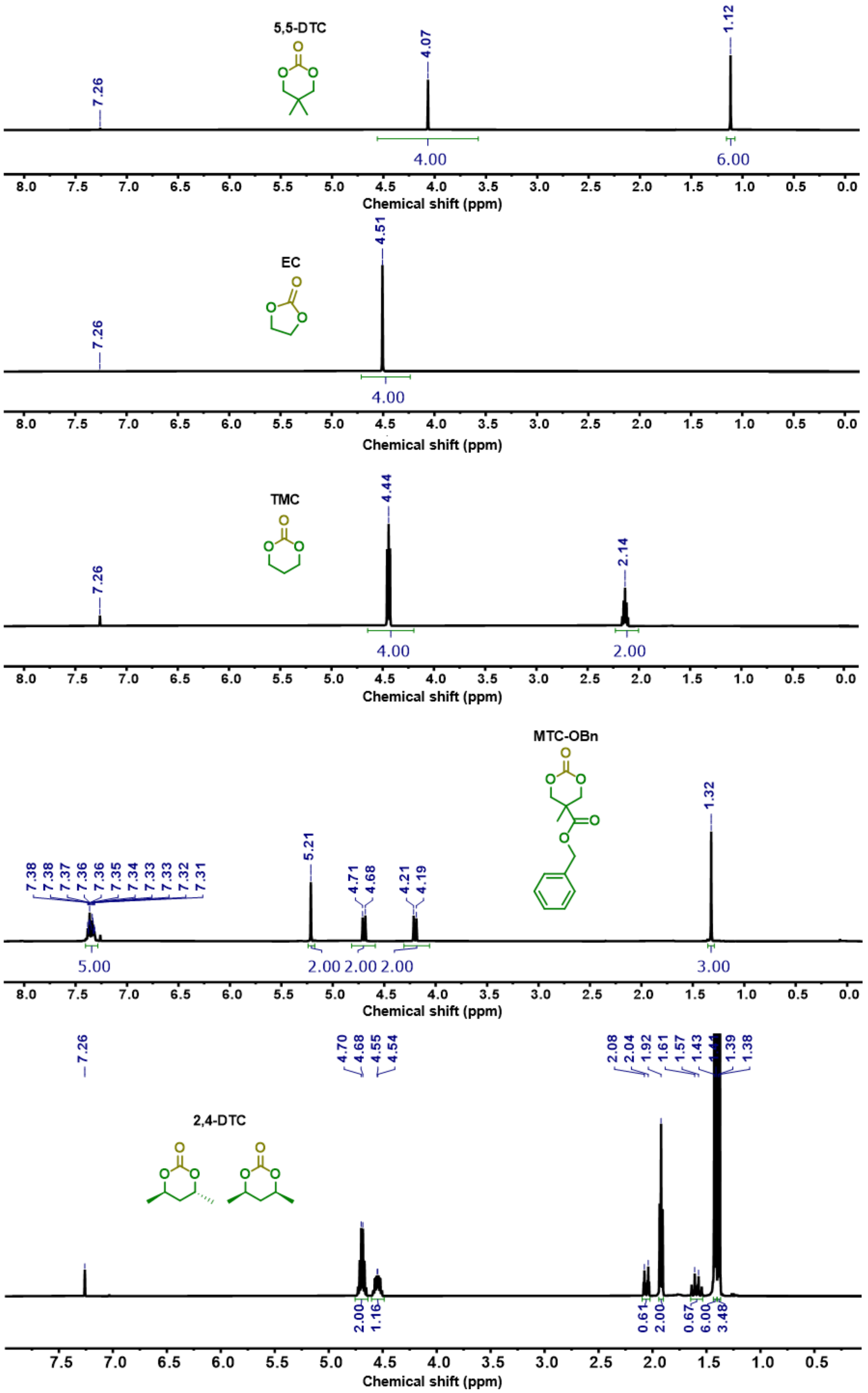

Figure S31. ^{1}H NMR ($CDCl_3$) of isolated cyclic carbonates from $P1_{Zn}$ chemical recycling. Products were isolated by dissolving the crude reaction mixture in DCM and washing with 1 M HCl. The organic layer was then dried over $MgSO_4$. TMC, 5,5-DTC and MTC-OBn were obtained as white solids. EC and 2,4-DTC were isolated as colourless liquids. TMC was of sufficient purity for repolymerization to comparable M_n (196 kg mol^{-1}) and $Đ$ (1.38) to **P3**.

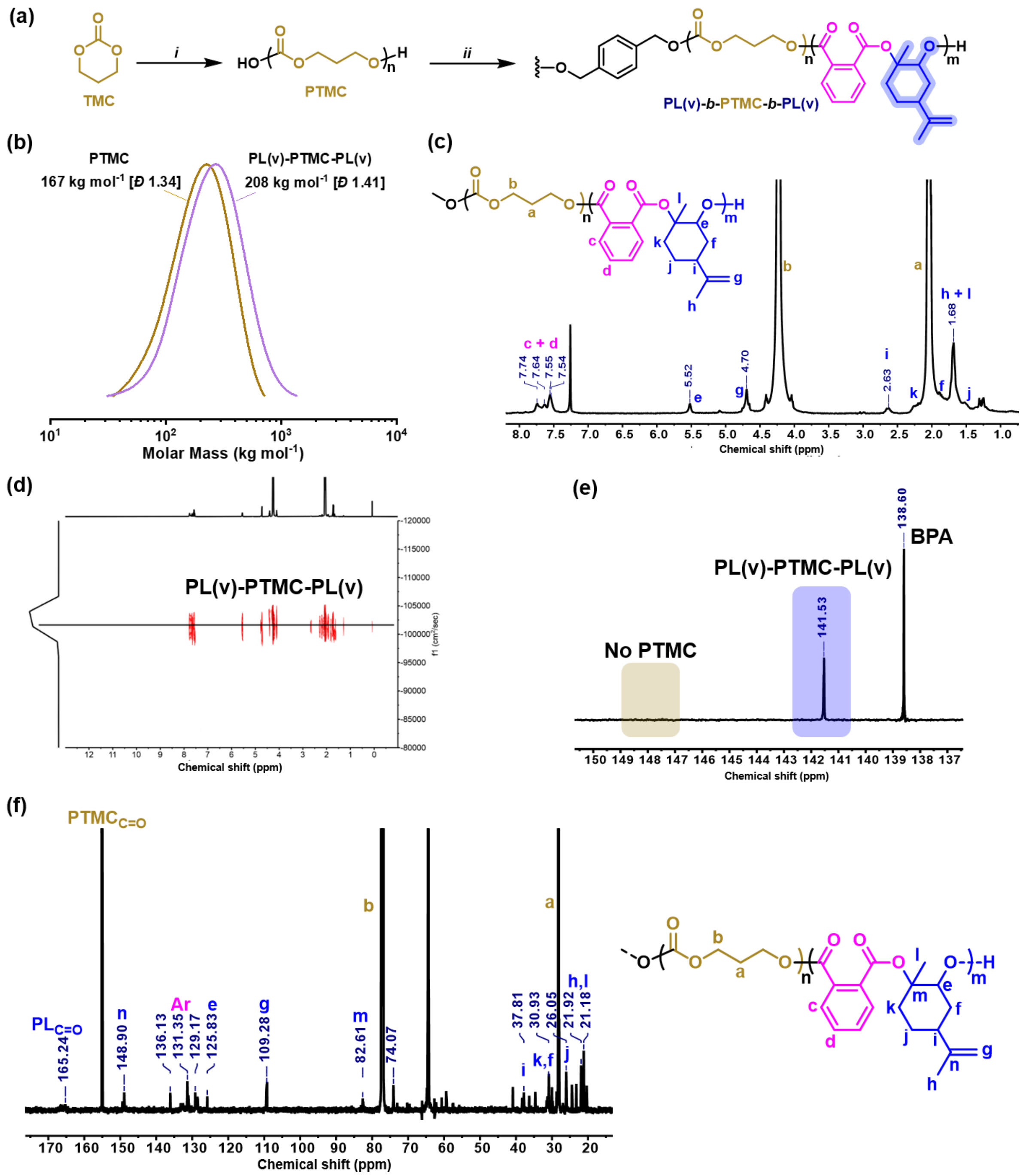

Figure S32. PL(v)-PTMC-PL(v) synthesis and characterization. (a) Reaction Scheme. *i* = 1 M $[TMC]_0$ in THF, RT, 0.5 h. *ii* = Addition of PA (400 equiv.) and the commercial mixture of *cis*- and *trans*- LO (600 equiv.). The reaction was conducted at 140 °C and reached 91% PA conversion (8.05-7.80 ppm) to PA/LO polyester (7.54-7.74 ppm) after 5 days. (b) ^{1}H NMR ($CDCl_3$) of isolated triblock polymer, **P4** (precipitated into diethyl ether). Resonances attributed to PL(v) are consistent with previous literature reports.[14] (c) SEC of purified **P4** and PTMC aliquot taken before adding PA/LO. (d) DOSY of **P4**. (e) $^{31}P\{^{1}H\}$ NMR. End-group analysis of isolated **P4**. (f) $^{13}C\{^{1}H\}$ NMR ($CDCl_3$) of **P4**. Only one regioisomer is shown for clarity.

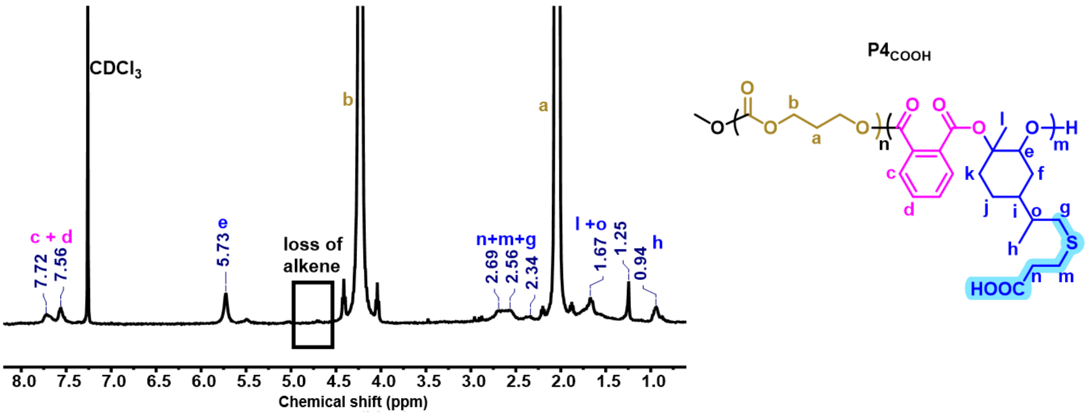

Figure S33. ^{1}H NMR ($CDCl_3$) of **$P4_{COOH}$** after thiol-ene reaction (as described above for **P1**-**P3**).

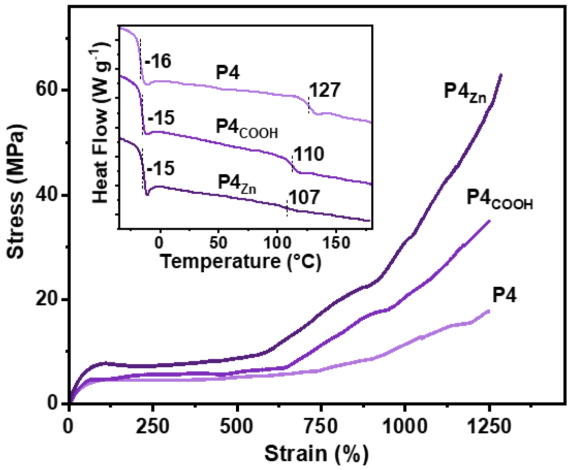

Figure S34. Representative tensile data for P_4, $P4_{COOH}$ and $P4_{Zn}$. Inset. DSC analysis.

References

[1] A. Spyros, D. S. Argyropoulos, R. H. Marchessault, *Macromolecules* **1997**, *30*, 327-329.

[2] a) J. Filik, A. W. Ashton, P. C. Y. Chang, P. A. Chater, S. J. Day, M. Drakopoulos, M. W. Gerring, M. L. Hart, O. V. Magdysyuk, S. Michalik, A. Smith, C. C. Tang, N. J. Terrill, M. T. Wharmby, H. Wilhelm, *J. Appl. Crystallogr.* **2017**, *50*, 959-966; b) B. R. Pauw, A. J. Smith, T. Snow, N. J. Terrill, A. F. Thünemann, *J. Appl. Crystallogr.* **2017**, *50*, 1800-1811.

[3] G. L. Gregory, M. Ulmann, A. Buchard, *RSC Adv.* **2015**, *5*, 39404-39408.

[4] a) G. S. Sulley, G. L. Gregory, T. T. D. Chen, L. Peña Carrodeguas, G. Trott, A. Santmarti, K.-Y. Lee, N. J. Terrill, C. K. Williams, *J. Am. Chem. Soc.* **2020**, *142*, 4367-4378; b) C. Romain, J. A. Garden, G. Trott, A. Buchard, A. J. P. White, C. K. Williams, *Chem. Eur. J.* **2017**, *23*, 7367-7376.

[5] a) A. Watts, N. Kurokawa, M. A. Hillmyer, *Biomacromolecules* **2017**, *18*, 1845-1854; b) C. J. Stubbs, J. C. Worch, H. Prydderch, Z. Wang, R. T. Mathers, A. V. Dobrynin, M. L. Becker, A. P. Dove, *J. Am. Chem. Soc.* **2022**, *144*, 1243-1250.

[6] J. Yang, F. Liu, L. Yang, S. Li, *Eur. Polym. J.* **2010**, *46*, 783-791.

[7] E. Bat, T. G. van Kooten, J. Feijen, D. W. Grijpma, *Acta biomaterialia* **2011**, *7*, 1939-1948.

[8] a) Z. Zhang, D. W. Grijpma, J. Feijen, *Macromol. Chem. Phys.* **2004**, *205*, 867-875; b) J.-H. Kim, J. H. Lee, *Polym. J.* **2002**, *34*, 203-208.

[9] G. L. Gregory, G. S. Sulley, L. P. Carrodeguas, T. T. D. Chen, A. Santmarti, N. J. Terrill, K.-Y. Lee, C. K. Williams, *Chem. Sci.* **2020**, *11*, 6567-6581.

[10] M. T. Martello, M. A. Hillmyer, *Macromolecules* **2011**, *44*, 8537-8545.

[11] G.-W. Yang, G.-P. Wu, *ACS Sust. Chem. Eng.* **2019**, *7*, 1372-1380.

[12] M. Jia, D. Zhang, G. W. de Kort, C. H. R. M. Wilsens, S. Rastogi, N. Hadjichristidis, Y. Gnanou, X. Feng, *Macromolecules* **2020**, *53*, 5297-5307.

[13] R. C. Pratt, F. Nederberg, R. M. Waymouth, J. L. Hedrick, *Chem. Commun.* **2008**, 114-116.

[14] a) E. H. Nejad, A. Paoniasari, C. G. W. van Melis, C. E. Koning, R. Duchateau, *Macromolecules* **2013**, *46*, 631-637; b) H. K. Ryu, D. Y. Bae, H. Lim, E. Lee, K.-s. Son, *Polym. Chem.* **2020**, *11*, 3756-3761.
